# The predictive value of machine learning for mortality risk in patients with acute coronary syndromes: a systematic review and meta-analysis

**DOI:** 10.1186/s40001-023-01027-4

**Published:** 2023-10-20

**Authors:** Xiaoxiao Zhang, Xi Wang, Luxin Xu, Jia Liu, Peng Ren, Huanlin Wu

**Affiliations:** 1 Dongzhimen Hospital, Beijing University of Chinese Medicine, Beijing, China; 2 School of Life Science and Engineering, Southwest University of Science and Technology, Mianyang, China

**Keywords:** Acute coronary syndromes, Mortality, Predictive models, Machine learning, Meta-analysis, Systematic review

## Abstract

**Background:**

Acute coronary syndromes (ACS) are the leading cause of global death. Optimizing mortality risk prediction and early identification of high-risk patients is essential for developing targeted prevention strategies. Many researchers have built machine learning (ML) models to predict the mortality risk in ACS patients. Our meta-analysis aimed to evaluate the predictive value of various ML models in predicting death in ACS patients at different times.

**Methods:**

PubMed, Embase, Web of Science, and Cochrane Library were searched systematically from database establishment to March 12, 2022 for studies developing or validating at least one ML predictive model for death in ACS patients. We used PROBAST to assess the risk of bias in the reported predictive models and a random-effects model to assess the pooled C-index and accuracy of these models.

**Results:**

Fifty papers were included, involving 216 ML prediction models, 119 of which were externally validated. The combined C-index of the ML models in the validation cohort predicting the in-hospital mortality, 30-day mortality, 3- or 6-month mortality, and 1 year or above mortality in ACS patients were 0.8633 (95% CI 0.8467–0.8802), 0.8296 (95% CI 0.8134–0.8462), 0.8205 (95% CI 0.7881–0.8541), and 0.8197 (95% CI 0.8042–0.8354), respectively, with the corresponding combined accuracy of 0.8569 (95% CI 0.8411–0.8715), 0.8282 (95% CI 0.7922–0.8591), 0.7303 (95% CI 0.7184–0.7418), and 0.7837 (95% CI 0.7455–0.8175), indicating that the ML models were relatively excellent in predicting ACS mortality at different times. Furthermore, common predictors of death in ML models included age, sex, systolic blood pressure, serum creatinine, Killip class, heart rate, diastolic blood pressure, blood glucose, and hemoglobin.

**Conclusions:**

The ML models had excellent predictive power for mortality in ACS, and the methodologies may need to be addressed before they can be used in clinical practice.

**Supplementary Information:**

The online version contains supplementary material available at 10.1186/s40001-023-01027-4.

## Introduction

Acute coronary syndromes (ACS) are the unstable and progressive stages of coronary heart disease, including ST-segment elevation myocardial infarction (STEMI), non-ST-elevation myocardial infarction (NSTEMI), and unstable angina (UA) [1]. Although advances in early reperfusion therapy and adjuvant drug therapy have improved the prognosis of ACS patients, ACS remains the leading cause of death worldwide [2]. More than 5% of patients with ACS die in-hospital [3], even up to 26.7% in some subgroups [4], and up to 26.5% in long-term follow-up [5]. Appropriate management can significantly improve the prognosis of patients with ACS; thus, timely and accurate identification of mortality risk and early and appropriate risk stratification are essential.

Traditional risk stratification of ACS patients is based on risk scoring systems, of which the Global Registry of Acute Coronary Events (GRACE) risk score and Thrombolysis in Myocardial Infarction (TIMI) risk score are the most widely used mortality risk prediction tools [6, 7]. Although these risk scores have been validated and are generally accepted, they have limitations in current clinical practice. First, these risk scores were developed based on data from earlier randomized controlled trials. During that period, contemporary therapies for acute myocardial infarction (AMI) were not widely available, and drug eluting stents and newer generation antiplatelets were not introduced. Therefore, the predictive effect of these risk scores in current practice is questionable [8]. Second, these risk scores use only selective variables based on traditional statistical methods, inevitably limiting the number of predictors and thus the possibility of missing important information [9]. In addition, traditional risk scores focus on predicting short-term mortality, such as in-hospital, 14-day, and 30-day mortality, and less on long-term mortality risk [10]. Therefore, widespread interest has been in exploring more accurate and comprehensive mortality risk prediction models.

Machine learning (ML) is a subdiscipline of artificial intelligence that uses algorithms to identify patterns in large data sets with multiple variables that can be continuously improved with additional data, resulting in pattern algorithms that can predict various outcomes [11]. It constructs models based on test inputs and correlates all or some predictor variables with the results to make data-driven predictions or decisions [12]. In recent years, ML has been increasingly used in the medical field, especially in the cardiovascular field, as the availability of medical data continues to increase and computer analysis capabilities continue to improve. Emerging research indicates that the introduction of ML models as a clinical tool to accurately predict the risk of death in ACS patients has the great potential [13]. However, the performance of different models tends to vary, and it is unclear whether ML models have robust performance in predicting the risk of death in ACS. Therefore, we performed this systematic evaluation and meta-analysis.

## Methods

This meta-analysis was reported according to the criteria of Preferred Reporting Items for Systematic Reviews and Meta-Analyses (PRISMA 2020) [14]. Before the study initiation, the protocol was registered and approved in the PROSPERO International Prospective Systematic Evaluation Register (CRD42022322721).

### Retrieval strategy

We extensively searched PubMed, Embase, Web of Science, and Cochrane Library databases. Searches were conducted from database creation to March 12, 2022, with no language restrictions, to explore machine learning for predicting the risk of death in patients with ACS. We searched using a combination of MeSH (Medical Subject Headings) terms and free-text terms. We used three sets of search terms, each of which had at least one word to match:

1. “acute coronary syndromes” “myocardial infarction” “ST-segment elevation myocardial infarction” “non-ST-segment elevation myocardial infarction” and “unstable angina pectoris”;
2. “machine learning” “deep learning” “migration learning” “random forest” “artificial neural network” “support vector machine” “nomogram” “XGboost” “decision trees” and “predictive models”;
3. “death” “mortality” and survival”. (Additional file 1: Table S1 for specific retrieval strategy).

### Inclusion and exclusion criteria

The inclusion criteria were as follows:

1. Participants with a precise diagnosis of ACS, including STEMI, NSTEMI, and UA.
2. ML models and predictor variables were clearly described.
3. ML algorithms and predicted outcomes are provided. These outcome metrics focus on C-index, area under the curve (AUC), sensitivity, specificity, accuracy, confusion matrix, prediction model type, and critical predictors.

The exclusion criteria were as follows:

1. Conference abstracts, letters, editorials, systematic reviews or meta-analyses, consensus statements, and guidelines.
2. Studies where the time of death is unknown.
3. The main interest is in assessing risk factors without constructing a mortality risk prediction model.
4. Studies for which the full text is not available.

### Data extraction

Two reviewers (X–XZ and XW) constructed standardized forms based on the Checklist for critical Appraisal and data extraction for systematic Reviews of prediction Modelling Studies (CHARMS) [15]. They independently extracted data from original research reports. Extracted data included study characteristics (first author, year of publication, study type, sample source, number of participants and number of events, time to death or follow-up), ML characteristics (external validation method, variable selection method, model type, predictors included in the final model and their number), reported outcomes (C-index, AUC, sensitivity, specificity, and accuracy) and methodological information. In addition, if an article describes more than one model, we extract the data for each model separately. In articles examining the performance of the same predictive model across outcomes or multiple timepoints, we retained the predictive model for the mentioned outcome or timepoint as the primary analysis for the study.

### Quality assessment

We assessed the risk of bias in reported prediction models using PROBAST, risk of a bias assessment tool designed to systematically evaluate diagnostic or prognostic prediction models [16]. It contains 20 questions reflecting the quality bias of the original study from four domains: participants, predictors, outcomes, and statistical analysis. Each domain has several questions with responses of “yes” “probably yes” “no” “may or may not” or “no information” questions. A domain was considered low risk if it contained all questions with “yes” or “probably yes” answers, and the model was rated as low risk when all domains were considered low risk. A domain is regarded as high risk if it contains at least one question marked “no” or “probably no”. The model is considered high risk when at least one domain is regarded as high risk. A domain is considered an unclear risk if it contains at least one question indicated as uninformative. The overall risk of bias is considered unclear when at least one domain is considered unclear, and the other domains are at low risk of bias. Two investigators (X–XZ and LX–X) completed the assessment independently; discrepancies were discussed with a third party and resolved by consensus.

### Outcomes indicators

Our outcome indicators for this systematic evaluation are the C-index and accuracy of the mortality risk model. We can obtain the accuracy directly from the confusion matrix and indirectly from the sensitivity and specificity combined with the number of deaths and the total sample size.

### Data analysis

We performed a random-effects model-based meta-analysis to pool the C-index and accuracy of all included studies. We used the 95% confidence interval (CI) as the effect measure. For original studies that lacked confidence intervals or standard errors for the C-index, we estimated them according to the formula proposed by the relevant study [17]. The formula follows: c is the reported C-statistic, n is the number of observed events, m is the total number of non-events, and *m* ∗  = *n* ∗  = ½(*m* + *n*)-1. We used R4.2.0 (R Development Core Team, Vienna, http://www.R-project.org) in Metafor, Matrix, and Meta packages for all statistical analyses.

$$\mathbf{S}\mathbf{E}(\mathbf{c})\approx \sqrt{\frac{{\varvec{c}}\left(1-{\varvec{c}}\right)[1+\frac{{{\varvec{n}}}^{\mathbf{*}}\left(1-{\varvec{c}}\right)}{2-{\varvec{c}}}+\frac{{{\varvec{m}}}^{\mathbf{*}}{\varvec{c}}}{1+{\varvec{c}}}]}{{\varvec{m}}{\varvec{n}}}}$$

## Results

### Literature screening results

**Fig. 1:**
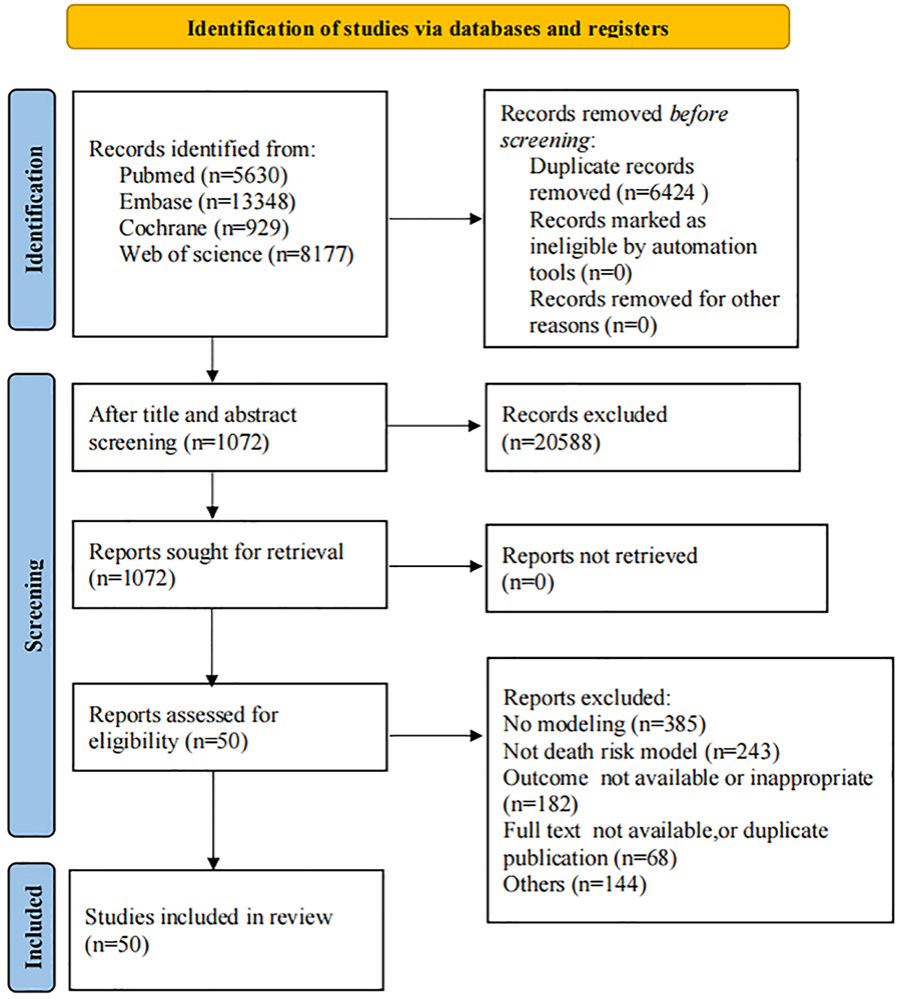
Flow chart of literature search and selection

The search identified a total of 28,084 articles, of which 6,424 were duplicates. Subsequently, 20,588 irrelevant articles were excluded after screening titles and abstracts, leaving 1072 articles for full-text screening. The final 50 eligible articles [18–67] evaluated the predictive effectiveness of the ML algorithm-based prediction model for predicting the risk of death in patients with ACS. Figure 1 shows the screening process.

### Eligible studies and the characteristics

All included studies were published as research articles in peer-reviewed medical journals from 2006 to 2022. We noted a broadly upward trend in publication, with 32 studies (64%) published since 2019, 18 studies (36%) published from 2006 to 2018, and no studies published before 2006. The 50 included studies involved 1,592,034 participants, and we calculated mortality using a single-arm meta-analysis: in-hospital mortality, 30-day mortality, 3- or 6-month mortality, and 1 year or more mortality were approximately 5.2% (95%CI 3.6–7.5), 6.5% (95%CI 4.9–8.5), 6.3% (95%CI 2.4–15.3), 8.3% (95%CI5.9–11.5), respectively. (Additional file 1: Fig. S1 for specific outcomes).These articles described a total of 216 ML models for predicting the risk of death in ACS, 119 of which were externally validated, involving logistic regression (LR), random forest (RF), artificial neural network (ANN), decision tree (DT), support vector machine (SVM), eXtreme Gradient Boosting (XGBoost), naive Bayesian (NB), adaptive boosting algorithm (AdaBoost), Bayesian Net classifier (BN), k-nearest neighbors algorithm (KNN), linear discriminant analysis (LDA), and more than 12 common prediction models. LR (*n* = 74) is the most commonly used modeling method, followed by RF (*n* = 35), which may be due to LR’s excellent performance in visualizing its model scores using nomograms. 21 studies had samples from public databases, including the Medical Information Mart for Intensive Care III database (MIMIC-III database) and multiple national acute myocardial infarction registry databases, 8 studies had samples from clinical trials, and 21 studies had samples from electronic health records. The key characteristics of the included studies are presented in Additional file 1: Table S2.

### Quality assessment of the selected studies

**Fig. 2:**
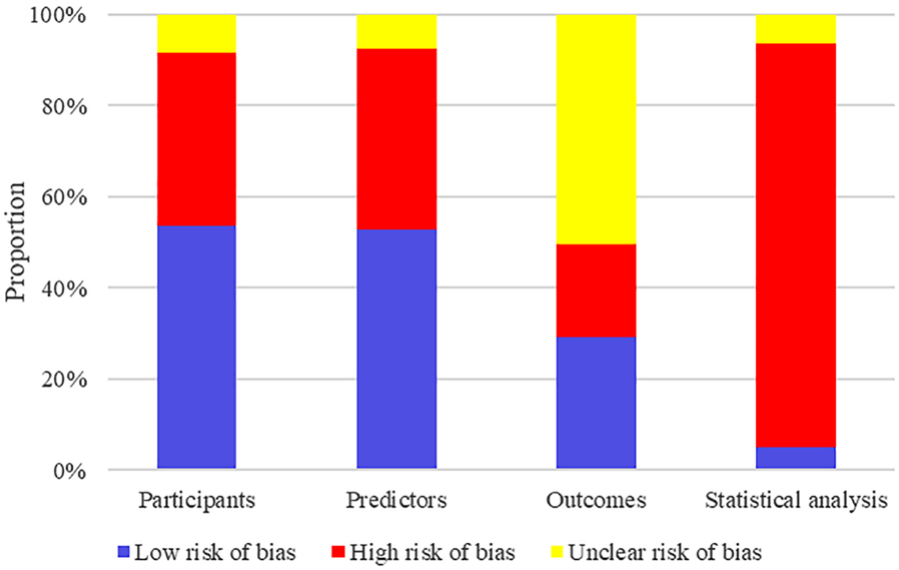
Risk of bias assessment (using PROBAST) based on four domains

We assessed the risk of bias for all developed or externally validated models according to PROBAST. Overall, ML models had a high risk of bias, mainly due to a large number of studies with subjects derived from retrospective case–control studies or without estimation of sample size according to the Events per variable (EPV) principle, thus not ensuring adequate sample size. In addition, 59 models were not externally validated. Moreover, the risk models covered mostly chose univariate + stepwise regression, LASSO regression, and the importance ranking of the models’ features (e.g., RF, SVM) in terms of feature selection. We summarized the risk of bias of the models by the four domains of PROBAST (Fig. 2).

### A meta-analysis of prediction model C-index and actual prediction accuracy

#### C-index for in-hospital mortality

The training cohort of 19 studies [18, 19, 22, 23, 26, 27, 29, 32, 34, 38, 40, 44–46, 54, 59–61, 64] developed 69 ML models predicting in-hospital mortality with a combined C-index of 0.8491 (95% CI 0.8337–0.8649). The validation cohort of 15 studies [18, 19, 23, 26–28, 32, 38, 45, 46, 54, 59–61, 64] reported 53 ML models predicting in-hospital mortality with a combined C-index of 0.8633 (95% CI 0.8467–0.8802) (Table 1) (Additional file 1: Fig. S2A).

**Table 1:** C-index for in-hospital mortality

No	Model	Training cohort		Validation cohort	
		Number of models	C-index (95% CI)	Number of models	C-index (95% CI)
1	LR	25	0.8603 [0.8330; 0.8886]	21	0.8576 [0.8284; 0.8878]
2	RF	9	0.8370 [0.7985; 0.8773]	12	0.8501 [0.8237; 0.8773]
3	ANN	10	0.8487 [0.8190; 0.8795]	2	0.9200 [0.9025; 0.9379]
4	DT	6	0.7957 [0.7390; 0.8567]	5	0.7691 [0.6674; 0.8864]
5	SVM	8	0.8491 [0.8048; 0.8958]	6	0.8737 [0.8551; 0.8927]
6	XGBoost	6	0.8953 [0.8526; 0.9401]	4	0.9295 [0.9094; 0.9500]
7	NB	1	0.8750 [0.8601; 0.8901]	2	0.8539 [0.8251; 0.8837]
8	KNN	1	0.7560 [0.7217; 0.7919]	1	0.9500 [0.9302; 0.9702]
9	Other	3	0.8416 [0.7825; 0.9051]		
10	Overall	69	0.8491 [0.8337; 0.8649]	53	0.8633 [0.8467; 0.8802]

#### C-index for 30-day mortality

The training cohort of 10 studies [20, 30, 41, 49–52, 64, 65] developed 30 ML models predicting 30-day mortality with a combined C-index of 0.8208 (95% CI 0.7940–0.8485). The validation cohort of 5 studies [30, 35, 50, 52, 64] reported 17 ML models predicting 30-day mortality with a combined C-index of 0.8296 (95% CI 0.8134–0.8462) (Table 2) (Additional file 1: Fig. S2B).

**Table 2:** C-index for 30-day mortality

No	Model	Training cohort		Validation cohort	
		Number of models	C-index (95% CI)	Number of models	C-index (95% CI)
1	LR	7	0.8249 [0.7952; 0.8556]	6	0.8298 [0.8135; 0.8464]
2	RF	3	0.9051 [0.8914; 0.9191]	5	0.8118 [0.7903; 0.8340]
3	ANN	3	0.8478 [0.8262; 0.8699]	2	0.8181 [0.7828; 0.8549]
4	DT	4	0.7831 [0.6766; 0.9063]	1	0.7800 [0.7701; 0.7901]
5	SVM	3	0.8919 [0.8752; 0.9089]	3	0.8852 [0.8635; 0.9075]
6	NB	4	0.7992 [0.7429; 0.8598]		
7	AdaBoost	1	0.8700 [0.8405; 0.9005]		
8	BN	2	0.7712 [0.7351; 0.8091]		
9	LDA	1	0.8410 [0.8069; 0.8766]		
10	Other	2	0.6400 [0.5944; 0.6890]		
11	Overall	30	0.8208 [0.7940; 0.8485]	17	0.8296 [0.8134; 0.8462]

#### C-index for 3- or 6-month mortality

Twenty ML models predicting 3- or 6-month mortality were developed in the training cohort of 6 studies [23, 24, 26, 49, 56, 66] with a combined C-index of 0.8227 (95% CI 0.8001–0.8460). The validation cohort of 5 studies [23, 24, 39, 56, 66] reported 8 ML models predicting 3- or 6-month mortality with a combined C-index of 0.8205 (95% CI 0.7881–0.8541) (Table 3) (Additional file 1: Fig. S2C).

**Table 3:** C-index for 3- or 6-month mortality

No	Model	Training cohort		Validation cohort	
		Number of models	C-index (95% CI)	Number of models	C-index (95% CI)
1	LR	7	0.8334 [0.8046; 0.8632]	3	0.8362 [0.7974; 0.8768]
2	RF	3	0.8304 [0.7542; 0.9143]	1	0.8500 [0.8450; 0.8550]
3	ANN	5	0.8436 [0.8041; 0.8850]	3	0.8054 [0.7340; 0.8838]
4	DT	1	0.7700 [0.7406; 0.8006]	1	0.7800 [0.7221; 0.8425]
5	SVM	2	0.7031 [0.6336; 0.7803]		
6	XGBoost	2	0.8119 [0.7599; 0.8675]		
7	Overall	20	0.8227 [0.8001; 0.8460]	8	0.8205 [0.7881; 0.8541]

#### C-index for 1 year or more mortality

The training cohort of 19 studies [23, 25–27, 31, 33, 36, 37, 42, 43, 47, 48, 52, 55, 57, 62–64, 67] developed 58 ML models predicting 1 year or more mortality with a combined C-index of 0.8352 (95% CI 0.8214–0.8493). The validation cohort of 11 studies [21, 23, 25, 26, 33, 42, 47, 48, 52, 57, 64] reported 41 ML models predicting 1 year or more mortality with a combined C-index of 0.8197 (95% CI 0.8042–0.8354) (Table 4) (Additional file 1: Fig. S2D).

**Table 4:** C-index for 1 year or more mortality

No	Model	Training cohort		Validation cohort	
		Number of models	C-index (95% CI)	Number of models	C-index (95% CI)
1	LR	23	0.8266 [0.8070; 0.8466]	11	0.8110 [0.7974; 0.8249]
2	RF	7	0.8623 [0.8284; 0.8975]	8	0.8127 [0.7898; 0.8362]
3	ANN	5	0.8172 [0.7804; 0.8557]	6	0.8454 [0.7989; 0.8946]
4	DT	8	0.8281 [0.7865; 0.8719]	3	0.7868 [0.7383; 0.8384]
5	SVM	7	0.8195 [0.7814; 0.8594]	6	0.7972 [0.7491; 0.8484]
6	XGBoost	3	0.8619 [0.8051; 0.9226]	2	0.8075 [0.7776; 0.8385]
7	NB	2	0.8930 [0.8621; 0.9249]		
8	AdaBoost	1	0.9100 [0.9001; 0.9201]	3	0.8905 [0.8248; 0.9615]
9	KNN	1	0.7840 [0.7498; 0.8198]		
10	Other	1	0.9240 [0.9190; 0.9290]	2	0.8869 [0.8581; 0.9166]
11	Overall	58	0.8352 [0.8214; 0.8493]	41	0.8197 [0.8042; 0.8354]

#### Accuracy for in-hospital mortality

The accuracy of 42 ML models to predict in-hospital mortality was reported in the training cohort of 15 studies [18, 19, 22, 23, 25, 27, 34, 38, 40, 44, 53, 54, 59–61] with a combined accuracy of 0.8434 (95% CI 0.8166–0.8669). The validation cohort of 13 studies [18, 19, 26–28, 38, 46, 53, 54, 59–61, 64] reported the accuracy of 45 ML models to predict in-hospital mortality with a combined accuracy of 0.8569 (95% CI 0.8411–0.8715) (Table 5) (Additional file 1: Fig. S2E).

**Table 5:** Accuracy for in-hospital mortality

No	Model	Training cohort		Validation cohort	
		Number of models	Accuracy (95% CI)	Number of models	ACC (95% CI)
1	LR	15	0.8302 [0.7800; 0.8709]	16	0.8487 [0.8250; 0.8697]
2	RF	6	0.8106 [0.7462; 0.8617]	12	0.8881 [0.8639; 0.9084]
3	ANN	3	0.8864 [0.7927; 0.9409]	2	0.8651 [0.8364; 0.8893]
4	DT	4	0.8824 [0.8094; 0.9299]	2	0.8464 [0.7164; 0.9232]
5	SVM	4	0.7902 [0.7333; 0.8376]	6	0.8199 [0.7772; 0.8559]
6	XGBoost	6	0.8679 [0.8033; 0.9136]	4	0.8505 [0.8332; 0.8663]
7	NB	1	0.7984 [0.7892; 0.8073]	2	0.7615 [0.7342; 0.7869]
8	KNN			1	0.9112 [0.8973; 0.9233]
9	Other	3	0.8800 [0.7551; 0.9458]		
10	Overall	42	0.8434 [0.8166; 0.8669]	45	0.8569 [0.8411; 0.8715]

#### Accuracy for 30-day mortality

The training cohort of 4 studies [20, 51, 52, 65] reported the accuracy of 17 ML models to predict 30-day mortality with a combined accuracy of 0.8257 (95% CI 0.7694–0.8707). The validation cohort of 2 studies [30, 64] reported the accuracy of 11 ML models to predict 30-day mortality with a combined accuracy of 0.8282 (95% CI 0.7922–0.8591) (Table 6) (Additional file 1: Fig. S2F).

**Table 6:** Accuracy for 30-day mortality

No	Model	Training cohort		Validation cohort	
		Number of models	Accuracy (95% CI)	Number of models	ACC (95% CI)
1	LR	4	0.7893 [0.6364; 0.8891]	3	0.8095 [0.7946; 0.8236]
2	RF	1	0.8429 [0.8289; 0.8560]	5	0.8533 [0.7843; 0.9029]
3	ANN	1	0.7669 [0.7623; 0.7714]		
4	DT	3	0.8209 [0.6871; 0.9054]		
5	SVM			3	0.8014 [0.7862; 0.8157]
6	NB	3	0.8845 [0.8104; 0.9321]		
7	AdaBoost	1	0.8088 [0.7937; 0.8230]		
8	BN	2	0.9212 [0.9068; 0.9335]		
9	Other	2	0.6536 [0.5015; 0.7797]		
10	Overall	17	0.8257 [0.7694; 0.8707]	11	0.8282 [0.7922; 0.8591]

#### Accuracy for 3- or 6-month mortality

The training cohort of 3 studies [23, 24, 26] reported the accuracy of 15 ML models to predict 3- or 6-month mortality, with a combined accuracy of 0.7089 (95%CI 0.6737–0.7418). The validation cohort of 1 study [24] reported the accuracy of 2 ML models to predict 3- or 6-month mortality, with a combined accuracy of 0.7303 (95%CI 0.7184–0.7418) (Table 7) (Additional file 1: Fig. S2G).

**Table 7:** Accuracy for 3- or 6-month mortality

No	Model	Training cohort		Validation cohort	
		Number of models	Accuracy (95%CI)	Number of models	ACC (95% CI)
1	LR	5	0.6977 [0.6622; 0.7310]	1	0.7218 [0.7069; 0.7363]
2	RF	2	0.7440 [0.6572; 0.8151]		
3	ANN	3	0.7079 [0.6674; 0.7454]	1	0.7387 [0.7240; 0.7529]
4	DT	1	0.6969 [0.6824; 0.7110]		
5	SVM	2	0.7233 [0.4934; 0.8752]		
6	XGBoost	2	0.6937 [0.6446; 0.7387]		
7	Overall	15	0.7089 [0.6737; 0.7418]	2	0.7303 [0.7184; 0.7418]

#### Accuracy for 1 year or more mortality

The training cohort of 12 studies [23, 26, 31, 33, 36, 37, 43, 48, 52, 55, 62, 63] reported the accuracy of 31 ML models predicting 1 year or more mortality with a combined accuracy of 0.7697 (95% CI 0.7360–0.8002). The validation cohort of 7 studies [21, 26, 33, 47, 48, 57, 64] reported the accuracy of 33 ML models predicting 1 year or more mortality with a combined accuracy of 0.7837 (95%CI 0.7455–0.8175) (Table 8) (Additional file 1: Fig. S2H).

**Table 8:** Accuracy for 1 year or more mortality

No	Model	Training cohort		Validation cohort	
		Number of models	Accuracy (95%CI)	Number of models	ACC (95% CI)
1	LR	12	0.7410 [0.7005; 0.7778]	8	0.7433 [0.7128; 0.7717]
2	RF	4	0.7982 [0.6922; 0.8744]	8	0.8032 [0.7253; 0.8632]
3	ANN	3	0.7481 [0.7252; 0.7697]	4	0.7995 [0.7004; 0.8718]
4	DT	4	0.7805 [0.6733; 0.8598]	1	0.7580 [0.7521; 0.7638]
5	SVM	2	0.6147 [0.5908; 0.6381]	6	0.7105 [0.6070; 0.7959]
6	XGBoost	3	0.8261 [0.7011; 0.9058]	2	0.7431 [0.6838; 0.7946]
7	NB	1	0.8698 [0.8499; 0.8874]		
8	AdaBoost	1	0.8657 [0.8603; 0.8709]	2	0.8261 [0.7392; 0.8884]
9	KNN	1	0.8304 [0.8085; 0.8502]		
10	Other			2	0.9370 [0.9208; 0.9501]
11	Overall	31	0.7697 [0.7360; 0.8002]	33	0.7837 [0.7455; 0.8175]

### Predictive variables for risk of death in ACS

**Fig. 3:**
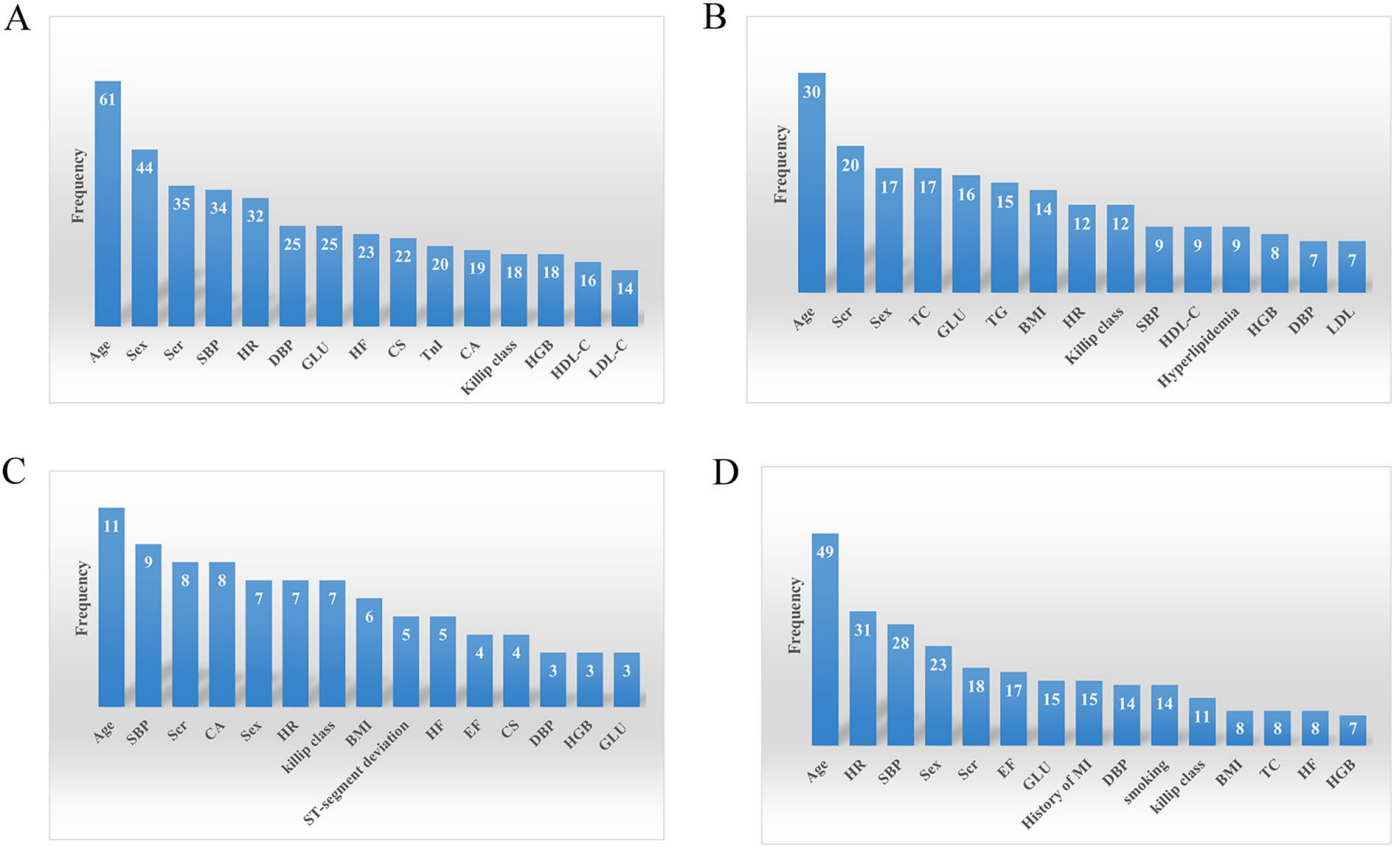
**A** variables of in-hospital mortality; **B** variables of 30-day mortality; **C** variables of 3- or 6-month mortality; **D** variables of 1 year or more mortality

To clarify the variables with the greatest predictive power in ML models, the data were further examined and counted, and 27 ML prediction models (10.96%) were found not to specify the predictors used by the model. Age was the most widely used predictor for short-term and long-term mortality prediction. Sex, systolic blood pressure, heart rate, serum creatinine, Killip classification, diastolic blood pressure, glucose, and hemoglobin were important predictors, all ranking in the top 15 predictor variables. We listed the most common predictors of ML models used to predict different times of death in ACS patients (Fig. 3, top 15).

## Discussion

Our systematic review included 50 original studies and reported 216 ML mortality risk prediction models constructed based on large samples. We found that (1) ML models predicting death in ACS patients at different times showed a relatively excellent prediction with a good composite C-index and accuracy; (2) the specific type of model and the variables included in the model severely affected the prediction of the model, with specific ML models predicting death in ACS patients showing excellent performance; (3) age, sex, systolic blood pressure, serum creatinine, Killip classification, heart rate, diastolic blood pressure, blood glucose, and hemoglobin were commonly used predictors, with age being the most commonly used and important predictor variable described.

ACS is a leading cause of death worldwide, and rapid identification of high-risk patients remains unmet clinical needs. For this purpose, several risk scores have been developed, among which the GRACE risk score is one of the best validated predictive tools. However, this score was developed based on traditional statistical methods that were useful and robust [13] but had inherent limitations. This limitation limits their ability to handle large data sets with multiple variables and samples [68]. The chosen predictor variables are run the same way for each individual and uniformly across the range [13]. There are non-linear relationships and complex interactions between ACS risk factors, and large population-level studies can provide critical insights into ACS risk factors [69]. Therefore, the inherent characteristics of traditional statistical methods may lead to low model predictive power. ML is an area of artificial intelligence that is part of a broader approach to data analysis [68]. Unlike traditional predictive models that use selected variables for computation, ML algorithms can easily combine many variables while capturing complex relationships between variables [69, 70]; and search for the best fit randomly or deterministically according to different algorithms ^13^ to construct robust prediction models. Most importantly, ML algorithms can better describe the complexity and unpredictability of human physiology in many cases [70]. These advantages make ML technology suitable for the medical field, especially for outcome prediction. Recent studies have shown that ML algorithms outperform traditional statistical modeling methods [13]. Our present systematic review supports the above view, showing that the ML algorithm-based prediction model has a more desirable integrated C-index and accuracy.

To visualize the contribution of each characteristic to the risk of occurrence of death in ACS, we calculated the importance of each characteristic. We identified nine variables commonly used to predict short- and long-term mortality in patients with ACS, including five variables from the GRACE score (age, systolic blood pressure, serum creatinine, Killip classification, heart rate) and four new variables (sex, diastolic blood pressure, blood glucose, and hemoglobin). These parameters describe non-modifiable risk factors and different pathophysiological contexts, such as hemodynamics, cardiovascular metabolism, and cardiomyocyte injury. Existing theories and studies also support these risk factors. Age was a well-established predictor and the most commonly used predictor variable found in our current study. Age-related pathological changes in the vascular system play a key role in morbidity and mortality in the elderly. As we age, changes in cardiovascular structure, function, and phenotype accelerate the progression of coronary artery disease, so older patients have more severe lesions and a higher risk of death [71].

There are gender differences in the outcomes of ACS patients. Studies have consistently shown that women with ACS have poorer short- and long-term outcomes than men, with a disproportionate risk of death [72, 73]. Female patients with ACS have different risk factor profiles and clinical presentations than male patients. In general, female ACS patients have a higher prevalence of cardiovascular risk factors, such as diabetes, hypertension, and psychosocial risk factors, such as depression [74]; more atypical symptoms, such as neck pain, fatigue, and dyspnea [75]; longer duration of ischemia due to pre-hospital delays, evidence-based diagnosis and inadequate treatment [76]; as well as a high rate of complications during hemodynamic reconstruction [77]. All of the above contribute to the high risk of death in women with ACS.

Independent of pre-existing metabolic dysregulation in diabetes, hyperglycemia at admission to ACS is associated with poor outcomes, regardless of diabetes status [78]. The mechanisms underlying the association between hyperglycemia and increased mortality in ACS patients are multifactorial. Increased local and systemic inflammatory responses [79], altered platelet function and thrombo-fibrinolytic system [80], increased oxidative stress [81], endothelial dysfunction [82], arrhythmic tendencies [83], and impaired myocardial contractility [84], all ultimately lead to increased atherosclerotic burden and plaque instability, and an increased risk of death.

We found hemoglobin to be a significant predictor of death in ACS. Recent studies have consistently shown that anemia on admission is always associated with poorer outcomes in ACS, as evidenced by increased mortality at different observed timepoints [85, 86]. An imbalance in myocardial oxygen supply and demand is necessary to develop ACS. Low hemoglobin levels worsen the myocardial ischemic injury by reducing the oxygen supply to the damaged myocardium. In contrast, increased myocardial oxygen demand exacerbates this imbalance due to the need for higher cardiac output to maintain adequate systemic oxygen supply [87]. Other plausible explanations are the reduced number of functionally impaired peripheral endothelial progenitor cells and impaired vascular healing capacity in ACS patients with low hemoglobin levels [88]. In addition, studies have shown that the inflammatory factor C-reactive protein(CRP) is negatively correlated with hemoglobin levels in patients with ACS, which may further increase the risk of death [89].

The present study found that LR was currently the most widely used modeling method in ACS mortality risk prediction models. It performed better in mortality risk models across time and even sometimes better than others, indicating the importance of valid predictors. Therefore, the development or updating of prediction models should be inclined to incorporate valid, easily collected, minimally invasive predictors.

### Limitations and strengths

Although the current results indicate that the predictive ability of ML models appears satisfactory, there are some methodological flaws or limitations to the inclusion of the original study. First, the ML models with different times of death were combined separately in the current study, and their discriminatory performance was assessed according to C-index and accuracy. Still, most risk models were not constructed with overfitting in mind. Second, a large amount of risk model data was derived from retrospective case–control studies and primarily generated training and validation cohorts at a certain ratio (e.g., 7/3) without external validation using an utterly new validation cohort. In addition, the modeling methods of most studied ML models are not clearly described, such as insufficient disclosure of information on hyperparameter tuning and external validation of ML algorithms. The development of predictive models helps in clinical decision-making and resource allocation. However, the risk of bias, reproducibility, and potential usefulness of predictive models can only be fully assessed if the modeling steps of predictive models are adequately and clearly reported [90]. The transparent reporting of a multivariable prediction model for individual prognosis or diagnosis (TRIPOD) statement presents a list of 22 items, thereby increasing the transparency of predictive modeling studies^91^. We strongly recommend detailed and standardized reporting of predictive models according to the TRIPOD statement, which not only helps to improve the quality of ML models but also helps to assess their reliability and increase their credibility.

Although we acknowledge the limitations of the original study, we believe that our meta-analysis still has some merit and clinical relevance. First, this is the first meta-analysis to systematically assess the predictive value of ML models for death at different times in patients with ACS. Second, we ranked the model variables of the original study according to importance, providing the most valuable variables for predicting ACS death, complementing variables not included in the GRACE risk score, which can guide further development of mortality risk models. In addition, the original studies included in our systematic review used different authoritative databases, including the MIMIC-III database and multiple national registry databases for acute myocardial infarction. The extensive use of different databases adds to some extent to the reliability of our results.

## Conclusion

Risk stratification of ACS patients is crucial for the early identification of high-risk patients to provide effective interventions. The ML model is a good prediction tool for predicting the long-term and short-term mortality risk in ACS patients. The commonly used predictors were age, sex, systolic blood pressure, serum creatinine, Killip classification, heart rate, diastolic blood pressure, blood glucose, and hemoglobin. These can guide future risk scoring systems’ development or update.

### Supplementary Information

**Additional file 1: Table S1.** Specific retrieval strategy. **Table S2.** Key characteristics of the included studies. **Figure S1.** **A** In-hospital mortality; **B** 30-day mortality; **C** 3- or 6-month mortality; **D** 1 year or more mortality. **Figure S2**. **A** C-index for in-hospital mortality; **B** C-index for 30-day mortality; **C** C-index for 3- or 6-month mortality; **D** C-index for 1 year or more mortality; **E** Accuracy for in-hospital mortality; **F** Accuracy for 30-day mortality; **G** Accuracy for 3- or 6-month mortality. **H** Accuracy for 1 year or more mortality.

## Data Availability

The data that support the findings of this study are available from the corresponding author upon reasonable request.
