# Supplementary material for: The predictive value of machine learning for mortality risk in patients with acute coronary syndromes: a systematic review and meta-analysis
Source: Eur J Med Res. 2023 Oct 20;28:451. doi: 10.1186/s40001-023-01027-4 (PMC10588162; doi:10.1186/s40001-023-01027-4)
Supplement: Supplementary file 1 — Additional file 1: Table S1. Specific retrieval strategy. Table S2. Key characteristics of the included studies. Figure S1. A In-hospital mortality; B 30-day mortality; C 3- or 6-month mortality; D 1 year or more mortality. Figure S2. A C-index for in-hospital mortality; B C-index for 30-day mortality; C C-index for 3- or 6-month mortality; D C-index for 1 year or more mortality; E Accuracy for in-hospital mortality; F Accuracy for 30-day mortality; G Accuracy for 3- or 6-month mortality. H Accuracy for 1 year or more mortality. [file 40001_2023_1027_MOESM1_ESM.docx]

**Supplementary materials**

**Supplementary Table S1.** Specific retrieval strategy

| **Search number** | | **Query** |
| --- | --- | --- |
| **PubMed** | | |
| #1 | | "Acute Coronary Syndrome"[Mesh] |
| #2 | | (((((Acute Coronary Syndrome[Title/Abstract]) OR (Acute Coronary Syndromes[Title/Abstract])) OR (Coronary Syndrome, Acute[Title/Abstract])) OR (Coronary Syndromes, Acute[Title/Abstract])) OR (Syndrome, Acute Coronary[Title/Abstract])) OR (Syndromes, Acute Coronary[Title/Abstract]) |
| #3 | | #1 OR #2 |
| #4 | | "Myocardial Infarction"[Mesh] |
| #5 | | (((((((Myocardial Infarction[Title/Abstract]) OR (Infarction, Myocardial[Title/Abstract])) OR (Infarctions, Myocardial[Title/Abstract])) OR (Myocardial Infarctions[Title/Abstract])) OR (Myocardial Infarct[Title/Abstract])) OR (Infarct, Myocardial[Title/Abstract])) OR (Infarcts, Myocardial[Title/Abstract])) OR (Myocardial Infarcts[Title/Abstract]) |
| #6 | | #4 OR #5 |
| #7 | | "ST Elevation Myocardial Infarction"[Mesh] |
| #8 | | (((ST Elevation Myocardial Infarction[Title/Abstract]) OR (ST Segment Elevation Myocardial Infarction[Title/Abstract])) OR (ST Elevated Myocardial Infarction[Title/Abstract])) OR (STEMI[Title/Abstract]) |
| #9 | | #7 OR #8 |
| #10 | | "Non-ST Elevated Myocardial Infarction"[Mesh] |
| #11 | | (((((((((Non-ST Elevated Myocardial Infarction[Title/Abstract]) OR (Non ST Elevated Myocardial Infarction[Title/Abstract])) OR (NSTEMI[Title/Abstract])) OR (Non-ST-Elevation Myocardial Infarction[Title/Abstract])) OR (Infarction, Non-ST-Elevation Myocardial[Title/Abstract])) OR (Infarctions, Non-ST-Elevation Myocardial[Title/Abstract])) OR (Myocardial Infarction, Non-ST-Elevation[Title/Abstract])) OR (Myocardial Infarctions, Non-ST-Elevation[Title/Abstract])) OR (Non ST Elevation Myocardial Infarction[Title/Abstract])) OR (Non-ST-Elevation Myocardial Infarctions[Title/Abstract]) |
| #12 | | #10 OR #11 |
| #13 | | "Angina, Unstable"[Mesh] |
| #14 | | ((((((((((((((((Angina, Unstable[Title/Abstract]) OR (Anginas, Unstable[Title/Abstract])) OR (Unstable Anginas[Title/Abstract])) OR (Angina Pectoris, Unstable[Title/Abstract])) OR (Angina Pectori, Unstable[Title/Abstract])) OR (Unstable Angina[Title/Abstract])) OR (Angina at Rest[Title/Abstract])) OR (Angina, Preinfarction[Title/Abstract])) OR (Anginas, Preinfarction[Title/Abstract])) OR (Preinfarction Angina[Title/Abstract])) OR (Preinfarction Anginas[Title/Abstract])) OR (Myocardial Preinfarction Syndrome[Title/Abstract])) OR (Myocardial Preinfarction Syndromes[Title/Abstract])) OR (Preinfarction Syndrome, Myocardial[Title/Abstract])) OR (Preinfarction Syndromes, Myocardial[Title/Abstract])) OR (Syndrome, Myocardial Preinfarction[Title/Abstract])) OR (Syndromes, Myocardial Preinfarction[Title/Abstract]) |
| #15 | | #13 OR #14 |
| #16 | | #3 OR #6 OR #9 OR #12 OR #15 |
| #17 | | "Machine Learning"[Mesh] |
| #18 | | ((((((((((((machine learning[Title/Abstract]) OR (Deep learning[Title/Abstract])) OR (Prediction model[Title/Abstract])) OR (Transfer Learning[Title/Abstract])) OR (random forest[Title/Abstract])) OR (artificial neural network[Title/Abstract])) OR (ANN[Title/Abstract])) OR (Support vector machine[Title/Abstract])) OR (SVM[Title/Abstract])) OR (Nomogram[Title/Abstract])) OR (XGboost[Title/Abstract])) OR (Logistic[Title/Abstract])) OR (Decision tree[Title/Abstract]) |
| #19 | | #17 OR #18 |
| #20 | | "Death"[Mesh] |
| #21 | | ((Death[Title/Abstract]) OR (Mortality[Title/Abstract])) OR (Survival[Title/Abstract]) |
| #22 | | #20 OR #21 |
| #23 | | #16 AND #19 AND #22 |
| **Embase** | | |
| #1 | 'acute coronary syndrome'/exp | |
| #2 | 'acute coronary syndrome'/exp OR 'acute coronary syndrome' OR (acute AND coronary AND ('syndrome'/exp OR syndrome)) OR 'acute coronary syndromes':ab,ti OR 'coronary syndrome, acute':ab,ti OR 'coronary syndromes, acute':ab,ti OR 'syndrome, acute coronary':ab,ti OR 'syndromes, acute coronary':ab,ti | |
| #3 | #1 OR #2 | |
| #4 | 'heart infarction'/exp | |
| #5 | 'myocardial infarction'/exp OR 'myocardial infarction' OR (myocardial AND ('infarction'/exp OR infarction)) OR 'infarction, myocardial':ab,ti OR 'infarctions, myocardial':ab,ti OR 'myocardial infarctions':ab,ti OR 'myocardial infarct':ab,ti OR 'infarct, myocardial':ab,ti OR 'infarcts, myocardial':ab,ti OR 'myocardial infarcts':ab,ti | |
| #6 | #4 OR #5 | |
| #7 | 'st segment elevation myocardial infarction'/exp | |
| #8 | 'st elevation myocardial infarction'/exp OR 'st elevation myocardial infarction' OR (('st'/exp OR st) AND ('elevation'/exp OR elevation) AND myocardial AND ('infarction'/exp OR infarction)) OR 'st segment elevation myocardial infarction':ab,ti OR 'st elevated myocardial infarction':ab,ti OR stemi:ab,ti | |
| #9 | #7 OR #8 | |
| #10 | 'non st segment elevation myocardial infarction'/exp | |
| #11 | 'non-st elevated myocardial infarction'/exp OR 'non-st elevated myocardial infarction' OR ('non st' AND elevated AND myocardial AND ('infarction'/exp OR infarction)) OR 'non st elevated myocardial infarction':ab,ti OR nstemi:ab,ti OR 'non-st-elevation myocardial infarction':ab,ti OR 'infarction, non-st-elevation myocardial':ab,ti OR 'infarctions, non-st-elevation myocardial':ab,ti OR 'myocardial infarction, non-st-elevation':ab,ti OR 'myocardial infarctions, non-st-elevation':ab,ti OR 'non st elevation myocardial infarction':ab,ti OR 'non-st-elevation myocardial infarctions':ab,ti | |
| #12 | #10 OR #11 | |
| #13 | 'unstable angina pectoris'/exp | |
| #14 | 'angina, unstable'/exp OR 'angina, unstable' OR (('angina,'/exp OR angina,) AND unstable) OR 'anginas, unstable':ab,ti OR 'unstable anginas':ab,ti OR 'angina pectoris, unstable':ab,ti OR 'angina pectori, unstable':ab,ti OR 'unstable angina':ab,ti OR 'angina at rest':ab,ti OR 'angina, preinfarction':ab,ti OR 'anginas, preinfarction':ab,ti OR 'preinfarction angina':ab,ti OR 'preinfarction anginas':ab,ti OR 'myocardial preinfarction syndrome':ab,ti OR 'myocardial preinfarction syndromes':ab,ti OR 'preinfarction syndrome, myocardial':ab,ti OR 'preinfarction syndromes, myocardial':ab,ti OR 'syndrome, myocardial preinfarction':ab,ti OR 'syndromes, myocardial preinfarction':ab,ti | |
| #15 | #13 OR #14 | |
| #16 | #3 OR #6 OR #9 OR #12 OR #15 | |
| #17 | 'machine learning'/exp | |
| #18 | 'machine learning'/exp OR 'machine learning' OR (('machine'/exp OR machine) AND ('learning'/exp OR learning)) OR 'deep learning':ab,ti OR 'prediction model':ab,ti OR 'transfer learning':ab,ti OR 'random forest':ab,ti OR 'artificial neural network':ab,ti OR ann:ab,ti OR 'support vector machine':ab,ti OR svm:ab,ti OR nomogram:ab,ti OR xgboost:ab,ti OR logistic:ab,ti OR 'decision tree':ab,ti | |
| #19 | #17 OR #18 | |
| #20 | 'death'/exp | |
| #21 | 'death'/exp OR death OR mortality:ab,ti OR survival:ab,ti | |
| #22 | #20 OR #21 | |
| #23 | #16 AND #19 AND #22 | |
| **Cochrane** | | |
| #1 | MeSH descriptor: [Acute Coronary Syndrome] explode all trees | |
| #2 | (Acute Coronary Syndrome):ti,ab,kw OR (Acute Coronary Syndromes):ti,ab,kw OR (Coronary Syndrome, Acute):ti,ab,kw OR (Coronary Syndromes, Acute):ti,ab,kw OR (Syndrome, Acute Coronary):ti,ab,kw | |
| #3 | (Syndromes, Acute Coronary):ti,ab,kw | |
| #4 | #1 or #2 or #3 | |
| #5 | MeSH descriptor: [Myocardial Infarction] explode all trees | |
| #6 | (Myocardial Infarction):ti,ab,kw OR (Infarction, Myocardial):ti,ab,kw OR (Infarctions, Myocardial):ti,ab,kw OR (Myocardial Infarctions):ti,ab,kw OR (Myocardial Infarct):ti,ab,kw | |
| #7 | (Infarct, Myocardial):ti,ab,kw OR (Infarcts, Myocardial):ti,ab,kw OR (Myocardial Infarcts):ti,ab,kw | |
| #8 | #5 or #6 or #7 | |
| #9 | MeSH descriptor: [ST Elevation Myocardial Infarction] explode all trees | |
| #10 | (ST Elevation Myocardial Infarction):ti,ab,kw OR (ST Segment Elevation Myocardial Infarction):ti,ab,kw OR (ST Elevated Myocardial Infarction):ti,ab,kw OR (STEMI):ti,ab,kw | |
| #11 | #9 or #10 | |
| #12 | MeSH descriptor: [Non-ST Elevated Myocardial Infarction] explode all trees | |
| #13 | (Non-ST Elevated Myocardial Infarction):ti,ab,kw OR (Non ST Elevated Myocardial Infarction):ti,ab,kw OR (NSTEMI):ti,ab,kw OR (Non-ST-Elevation Myocardial Infarction):ti,ab,kw OR (Infarction, Non-ST-Elevation Myocardial):ti,ab,kw | |
| #14 | (Infarctions, Non-ST-Elevation Myocardial):ti,ab,kw OR (Myocardial Infarction, Non-ST-Elevation):ti,ab,kw OR (Myocardial Infarctions, Non-ST-Elevation):ti,ab,kw OR (Non ST Elevation Myocardial Infarction):ti,ab,kw OR (Non-ST-Elevation Myocardial Infarctions):ti,ab,kw | |
| #15 | #12 or #13 or #14 | |
| #16 | MeSH descriptor: [Angina, Unstable] explode all trees | |
| #17 | (Angina, Unstable):ti,ab,kw OR (Anginas, Unstable):ti,ab,kw OR (Unstable Anginas):ti,ab,kw OR (Angina Pectoris, Unstable):ti,ab,kw OR (Angina Pectori, Unstable):ti,ab,kw | |
| #18 | (Unstable Angina):ti,ab,kw OR (Angina at Rest):ti,ab,kw OR (Angina, Preinfarction):ti,ab,kw OR (Anginas, Preinfarction):ti,ab,kw OR (Preinfarction Angina):ti,ab,kw | |
| #19 | (Preinfarction Anginas):ti,ab,kw OR (Myocardial Preinfarction Syndrome):ti,ab,kw OR (Myocardial Preinfarction Syndromes):ti,ab,kw OR (Preinfarction Syndrome, Myocardial):ti,ab,kw OR (Preinfarction Syndromes, Myocardial):ti,ab,kw | |
| #20 | (Syndrome, Myocardial Preinfarction):ti,ab,kw OR (Syndromes, Myocardial Preinfarction):ti,ab,kw | |
| #21 | #16 or #17 or #18 or #19 or #20 | |
| #22 | MeSH descriptor: [Machine Learning] explode all trees | |
| #23 | (machine learning):ti,ab,kw OR (Deep learning):ti,ab,kw OR (Prediction model):ti,ab,kw OR (Transfer Learning):ti,ab,kw OR (random forest):ti,ab,kw | |
| #24 | (artificial neural network):ti,ab,kw OR (ANN):ti,ab,kw OR (Support vector machine):ti,ab,kw OR (SVM):ti,ab,kw OR (Nomogram):ti,ab,kw | |
| #25 | (XGboost):ti,ab,kw OR (Logistic):ti,ab,kw OR (Decision tree):ti,ab,kw | |
| #26 | #22 or #23 or #24 or #25 | |
| #27 | MeSH descriptor: [Death] explode all trees | |
| #28 | (Death):ti,ab,kw OR (Mortality):ti,ab,kw OR (Survival):ti,ab,kw | |
| #29 | #27 or #28 | |
| #30 | #4 or #8 or #11 or #15 or #21 | |
| #31 | #30 and #26 and #29 | |
| **Web of Science** | | |
| #1 | Acute Coronary Syndrome (Topic) or Acute Coronary Syndromes (Topic) or Coronary Syndrome, Acute (Topic) or Coronary Syndromes, Acute (Topic) or Syndrome, Acute Coronary (Topic) or Syndromes, Acute Coronary (Topic) | |
| #2 | Myocardial Infarction (Topic) or Infarction, Myocardial (Topic) or Infarctions, Myocardial (Topic) or Myocardial Infarctions (Topic) or Myocardial Infarct (Topic) or Infarct, Myocardial (Topic) or Infarcts, Myocardial (Topic) or Myocardial Infarcts (Topic) | |
| #3 | ST Elevation Myocardial Infarction (Topic) or ST Segment Elevation Myocardial Infarction (Topic) or ST Elevated Myocardial Infarction (Topic) or STEMI (Topic) | |
| #4 | Non-ST Elevated Myocardial Infarction (Topic) or Non ST Elevated Myocardial Infarction (Topic) or Non-ST-Elevation Myocardial Infarction (Topic) or Infarction, Non-ST-Elevation Myocardial (Topic) or Infarctions, Non-ST-Elevation Myocardial (Topic) or Myocardial Infarction, Non-ST-Elevation (Topic) or Myocardial Infarctions, Non-ST-Elevation (Topic) or Non ST Elevation Myocardial Infarction (Topic) or Non-ST-Elevation Myocardial Infarctions (Topic) or NSTEMI (Topic) | |
| #5 | Angina, Unstable (Topic) or Anginas, Unstable (Topic) or Unstable Anginas (Topic) or Angina Pectoris, Unstable (Topic) or Angina Pectori, Unstable (Topic) or Unstable Angina (Topic) or Angina at Rest (Topic) or Angina, Preinfarction (Topic) or Anginas, Preinfarction (Topic) or Preinfarction Angina (Topic) or Preinfarction Anginas (Topic) or Myocardial Preinfarction Syndrome (Topic) or Myocardial Preinfarction Syndromes (Topic) or Preinfarction Syndrome, Myocardial (Topic) or Preinfarction Syndromes, Myocardial (Topic) or Syndrome, Myocardial Preinfarction (Topic) or Syndromes, Myocardial Preinfarction (Topic) | |
| #6 | #1 OR #2 OR #3 OR #4 OR #5 | |
| #7 | machine learning (Topic) or Deep learning (Topic) or Prediction model (Topic) or Transfer Learning (Topic) or random forest (Topic) or artificial neural network (Topic) or ANN (Topic) or Support vector machine (Topic) or SVM (Topic) or Nomogram (Topic) or XGboost (Topic) or Logistic (Topic) or Decision tree (Topic) | |
| #8 | Death (Topic) or Mortality (Topic) or Survival (Topic) | |
| #9 | #6 AND #7 AND #8 | |

**Supplementary Table S2.** The key characteristics of the included studies

| **Study ID** | **Country** | **Study type** | **Disease type** | **Sample source** | **Age** | **Sex** | **Death sample of the training cohort** | **Total sample of the training cohort** | **External validation** | **Death sample of the validation cohort** | **Total sample of the validation cohort** | **Time to death or follow-up** |
| --- | --- | --- | --- | --- | --- | --- | --- | --- | --- | --- | --- | --- |
| Yudan Wang 2022 | China | retrospective study | STEMI | 39centers | T: (63.3 ± 12.7)  V: (62.1 ± 12.8) | NA | 132 | 396 | yes | 91 | 459 | in-hospital mortality |
| BlancaVázquez 2021 | Mexico | retrospective study | ACS (STEMI, NSTEMI) | MIMIC-III database | STEMI: (67.26± 13.86)  NSTEMI: (72.29 ± 13.38) | STEMI: Female 460 (35%)  Male 839 (65%),  NSTEMI: Female 1176 (42%)  Male 1644 (58%) | 278 | STEMI-1039  NSTEMI-2256 | yes | STEMI-18  NSTEMI-52 | STEMI-260  NSTEMI-564 | in-hospital mortality |
| Roni Shouval 2017 | Israel | retrospective study | STEMI | ACSIS | 60.5 ± 12.4 | Female 497 (17.9%)  Male 2285 (82.1%) | 124 | 2782 | none | — | — | in-hospital mortality |
| Syed Waseem Abbas Sherazi 2020 | Korea | retrospective study | ACS | KAMIR | 62.19 ± 12.54 | Female 2348 (28.5%)  Male 5890 (71.5%) | 305 | 6606 | yes | 90 | 1621 | 1-year mortality |
| Konrad Pieszko 2018 | Poland | retrospective study | ACS (STEMI, NSTE-ACS or UA) | EMR | Significant lesion: 65.8,  No significant lesion: 67,  In-hospital death: 75.8,  No in-hospital death: 66 | Significant lesion: Female 1488 (30%)  Male 3342 (68%)  No significant lesion: Female 641 (35%)  Male 1138 (62%)  In-hospital death: Female 38 (39.2%)  Male 56 (57.7%)  No in-hospital death: Female 2091 (31%)  Male 4424 (66%) | 97 | 6769 | none |  |  | in-hospital mortality |
| Konrad Pieszko 2019 | Poland | retrospective study | ACS (STEMI, NSTEMI, UA) | EMR + national death registry | Survival:65.5 (59.4-73.0),  Death: 72.1 (64.4-79.8) | Survival: Female1379,  Male 2908,  Death: Female273,  Male 493 | in-hospital mortality-65  6-month mortality-233  1-year mortality-312 | 3975 | yes | in-hospital mortality-16  6-month mortality-59  1-year mortality-78 | 994 | in-hospital mortality  6-month mortality  1-year mortality |
| Jacek T Niedziela 2021 | Poland | retrospective study | STEMI | PL-ACS | Survival:62.9,  Death :72.6 | Survival: Female 5297 (31.7%)  Death: Female 458 (43.3%) | 630 | 10 675 | yes | 420 | 7118 | 6-month mortality |
| Paul D Myers 2017 | USA | retrospective study | NSTE-ACS | EMR | T: 63 (55 to 71),  V: 63 (54 to 72) | T: Female 1538 (35%),  V: Female 310 (36%) | 120 | 3516 | yes | 14 | 861 | 1-year mortality |
| Woojoo Lee 2021 | Korea | retrospective study | AMI (STEMI, NSTEMI) | KRAMI-RCC and KAMIR-NIH | STEMI: Survival (62.4 ± 12.5),  Death (73.9 ± 12.3),  NSTEMI: Survival (66.3 ± 12.5),  Death (76.5 ± 9.5) | Female: STEMI: Survival 895 (17.4%),  Death 135 (33.6%),  NSTEMI: Survival 2242 (28.0%),  Death 250 (40.7%) | STEMI: in-hospital mortality-211  3-month mortality-47  1-year mortality-88  NSTEMI: in-hospital mortality-215  3-month mortality-106  1-year mortality-235 | STEMI: in-hospital mortality-4443  3-month mortality-3918  1-year mortality-3922  NSTEMI: in-hospital mortality-6926  3-month mortality-6187  1-year mortality-6178 | yes | STEMI: in-hospital mortality-57  1-year mortality-46  NSTEMI: in-hospital mortality-61  1-year mortality-118 | STEMI: in-hospital mortality-5235  1-year mortality-5031  NSTEMI: in-hospital mortality-5924  1-year mortality-5622 | in-hospital mortality  3-month mortality  1-year mortality |
| D J Kurz 2009 | Switzerland | retrospective study | ACS | AMIS-Plus database | T:65.9,  V:66.1 | Male: T: 5415 (72.0%),  V: 2062 (72.2%) | in-hospital mortality-564  1-year mortality-176 | in-hospital mortality-7520  1-year mortality-1972 | yes | 357 | 5219 | in-hospital mortality  1-year mortality |
| Jun Ke 2022 | China | retrospective study | ACS | EMR | T: 67(60, 75),  V: 67(59, 75) | T: Female 1150 (25.3%),  Male 3387 (74.7%),  V: Female 467 (24.0%),  Male 1478 (76.0%) | 85 | 4537 | yes | 37 | 1945 | in-hospital mortality |
| Jaroslav Hubacek 2006 | Canada | prospective cohort study | ACS | Train: prospective cohort study  Test: BCCR | 70-79y: 2363(23.5%),  ≥80y: 698(6.9%) | Female: 2681(26.7%) | 201 | 10050 | yes | — | 3259 | in-hospital mortality |
| Amir Hadanny 2021 | Israel | retrospective study | ACS | ACSIS and MINAP | T: (60.8±12.7),  V: (63.5±13.1) | Male: T:2038(82.3%),  V:16786(73.1%) | 110 | 2476 | yes | 989 | 22963 | 30-day mortality |
| Vasim Farooq 2013 | Netherlands | prospective cohort study | NSTE | ACUITY trial | 60.7±11.7 | Male 1774(67.5%) | 62 | 2627 | none | — | — | 1-year mortality |
| Irene R Dégano 2015 | Spain | retrospective study | ACS | EURHOBOP | T: AMI-67.7, PCI- 64.6  V: AMI-69.0, PCI-65.2 | Female T: AMI-3599 (30.9%),  PCI-2050 (24.8%),  V: AMI-17, 972 (33.2%),  PCI-6500 (25.2%) | AMI-868  PCI-278 | AMI-11631  PCI-8276 | yes | 5446 | 79830 | in-hospital mortality |
| Fabrizio D'Ascenzo 2021 | Italy | retrospective study | ACS | BleeMACS + RENAMI | T:64(54–73),V:68 (58–76) | T: Female 4363(22.0%), Male 15463(78.0%),  V: Female 953 (27.7%),  Male 2491 (72.3%) | 530 | 15861 | yes | 190 | 7409 | 1-year mortality |
| Shao-di Yan 2016 | China | retrospective study | ACS | EMR | 64.55± 10.66 | Male 1759 (78.9%) | 56 | 2229 | none | — | — | in-hospital mortality |
| Francisco Valente 2021 | Portugal | retrospective study | ACS | EMR | 64 (54–73) | Female 260 (23.4%),  Male 851 (76.6%) | 44 | 889 | yes | 11 | 222 | 30-day mortality |
| Ana T Timóteo 2014 | Portugal | prospective cohort study | ACS | EMR | 64 ± 13 | Male 1450(69.1%) | 203 | 2099 | none | — | — | 1-year mortality |
| Ana T Timóteo 2015 | Portugal | prospective cohort study | ACS | EMR | 64 ± 13 | Male 550(70%) | 61 | 787 | none | — | — | 1-year mortality |
| Liao Tan 2021 | China | retrospective study | AMI | eICU-CRD v2.0 + MIMIC-III | T: (65±44.13),  V: (66±91.13) | T: Female 989 (36.58%),  Male 1715 (63.42%),  V: Female 325 (31.68%),  Male 701 (68.32%) | 335 | 2704 | yes | 136 | 1026 | in-hospital mortality |
| Paul D Myers 2019 | USA | retrospective study | ACS | GRACE-ACS | T: 66.1 (55.7–75.8),  V: 68.2 (57.1–77.6) | T: Female 14038 (32.6%),  V: Female 2157 (33.9%) | 3078 | 43063 | yes | 719 | 6363 | 6-month mortality |
| Rohan Khera 2021 | USA | retrospective study | AMI | the American College of Cardiology Chest Pain-MI Registry | T:(65±14),V:(65±13) | Male:  T-369455 (65%),  V-125 747 (66%) | 24856 | 564918 | yes | 8381 | 190484 | in-hospital mortality |
| Zhengxing Huang 2018 | China | retrospective study | ACS | EMR | 63.18±12.18 | Male 2302 (70.9%) | 443 | 3464 | none | — | — | 30-day mortality |
| Dongze Li 2021 | China | retrospective study | AMI | EMR | T: Survival-(65.1 ± 13.0),  Death-(73.5 ± 10.0) | T: Male Survival-1208 (76.3%),  Death-110 (66.7%) | 165 | 1749 | yes | 82 | 870 | 2-year mortality |
| Truong H Hoang 2021 | Russia | prospective cohort study | AMI | EMR | 68.5 (58.7-76) | Female 90 (51.7%) | 35 | 174 | none | — | — | 18-month mortality |
| Jia Zhao 2021 | China | retrospective study | STEMI | EMR | 63.00 ±16 | Male 4198 (73.55%) | 116 | 4281 | none | 38 | 1427 | in-hospital mortality |
| Iman Yosefian 2015 | Iran | retrospective study | AMI | EMR | 61.34± 13.46 | Female 184 (30.3%). 423 (69.7%) | 204 | 607 | yes | 336 | 1000 | in-hospital mortality |
| Ni Wang 2022 | China | retrospective study | AMI | MIMIC-III+EMR | ≥60y: T-2408 (80.0%).  V-1131 (61.3%) | Male: T- 1855 (61.6%),  V-1343 (72.8%) | 245 | 3010 | yes | 132 | 1846 | in-hospital mortality |
| John Wallert 2017 | Sweden | prospective cohort study | MI | SWEDEHEART/RIKS-HIA | 68.8 ± 12.3 | Male: 33, 620 (64.7%) | 3426 | 31166 | yes | 2285 | 20777 | 2-year mortality |
| Alessia Vignoli 2019 | Italy | prospective cohort study | AMI | 6centers | Survival: 72 (62-80),  Death: 82 (78-83) | Female: Survival-278 (33.4%),  Death-67 (45.9%) | 40 | 120 | yes | 106 | 858 | 2-year mortality |
| Yu Tan 2021 | China | prospective cohort study | STEMI | EMR | 59.8±12.4 | Male 360 (81.1%) | 30-day mortality-13  6-month mortality-18 | 444 | none | — | — | 30-day mortality  6-month mortality |
| Ewout W Steyerberg 2014 | Netherlands | retrospective study | AMI | EMR | NA | NA | 1565 | 23034 | yes | 1286 | 17 796 | 30-day mortality |
| Salman I 2019 | Czech Republic | retrospective study | AMI | EMR | (23,94) | NA | 87 | 787 | none | — | — | 30-day mortality |
| Piros, P 2019 | Hungary | retrospective study | STEMI+NSTEMI | HUMIR | STEMI: (65±13.08)  NSTEMI: (69.5±12.26) | Male: STEMI-12448 (61%), NSTEMI-15829 (58%) | 30-day mortality-3701  1-year mortality-6547 | 33167 | yes | 30-day mortality-1587  1-year mortality-2808 | 14224 | 30-day mortality  1-year mortality |
| D. Mendes 2018 | Portugal | retrospective study | ACS | PSC | T:(66.06±13.86)  V-(66.39±13.38) | NA | 286 | 7717 | yes | 95 | 2385 | in-hospital mortality |
| Hend Mansoor 2017 | USA | retrospective study | STEMI | NIS | T:(68.3±14.5)  V-(69.3±14.4) | NA | 1064 | 9637 | yes | 3048 | 27369 | in-hospital mortality |
| Yi-Ming Li 2020 | China | prospective cohort study | STEMI | EMR | 63.76±12.92 | Male:975 (78.38%) | 185 | 1244 | none | — | — | 1-year mortality |
| Christien Kh Li 2020 | China | retrospective study | AMI | EMR | T:(71.08 ± 14.04) | T:Male 1393(65.5%) | 1424 | 2127 | yes | 828 | 1276 | 3-month mortality |
| Han Cheol Lee 2020 | Korea | prospective cohort study | AMI | KAMIR | T: Survival-(63±13)  Death-(73±11) | Male: Survival- 10504 (72.6%),  Death-526 (59.6%) | 989 | 15373 | yes | 343 | 6589 | 1-year mortality |
| Meng-Hsuen Hsieh 2019 | China | retrospective study | AMI | NHIRD | Survival:(64.5±13.6)  Death:(72.2±13.0) | Survival: Female 783(23.8%),  Male 2,513(76.2%),  Death: Female 40(32.0%),  Male 85(68.0%) | 125 | 3421 | yes | — | — | 30-day mortality |
| Yuhei Goriki 2020 | Japan | retrospective study | STEMI | EMR | T:(68.5 ± 12.6)  V-(68.9 ± 13.0) | Male: T- 493 (73.7%),  V-257 (71.8%) | 34 | 669 | yes | 23 | 358 | in-hospital mortality |
| Nan Gao 2020 | China | retrospective study | STEMI | EMR | T: Survival-(59.6 ± 11.4)  Death-(66.3 ± 13.3)  V: Survival-(60.0 ± 12.1)  Death-(66.0 ± 14.2) | Male: T: Survival-816 (76.0%),  Death-60 (63.2%),  V: Survival-207 (71.1%),  Death-18 (72.0%) | 95 | 1169 | yes | 25 | 316 | in-hospital mortality |
| Rui Fu 2018 | China | retrospective study | NSTEMI | CAMI | Survival: (64.92±11.98)  Death: (72.13±11.16) | Male: Survival-3,754 (69.1%),  Death-187 (54.7%) | 274 | 4332 | yes | 68 | 1443 | in-hospital mortality |
| Victoria A Brazhnik 2021 | Russia | prospective cohort study | ACS | ORACLE II Clinical Trials | T: (64.9±12.78) | Male: T-1120 (62.1%) | 228 | 1803 | yes | — | 429 | 1-year mortality |
| Theodor Baars 2018 | Germany | retrospective study | AMI | EMR | Survival:(64.37 ±0.65)  Death:(72.87 ±0.87) | Survival: Female 110(26.63%),  Male 303(73.37%),  Death: Female 53 (33.97%),  Male 103 (66.03%) | 156 | 569 | none | — | — | 6-year mortality |
| Firdaus Aziz 2021 | Malaysia | retrospective study | STEMI | NCVD-ACS | in-hospital mortality: (55.8 ± 11.5)  30-day mortality: (56.6 ± 11.7)  1-year mortality: (56.6 ± 11.6) | in-hospital mortality: Male 5417 (86.0%),  Female 882 (14.0%),  30-day mortality: Male 2681 (85.7%),  Female 448 (14.3%), 1-year mortality: Male 2533 (86.2),  Female 406 (13.8%) | in-hospital mortality-338  30-day mortality-252  1-year mortality-423 | in-hospital mortality-6299  30-day mortality-3130  1-year mortality-2939 | yes | in-hospital mortality-101  30-day mortality-75  1-year mortality-126 | in-hospital mortality-1889  30-day mortality-938  1-year mortality-880 | in-hospital mortality  30-day mortality  1-year mortality |
| Aziida, N 2021 | Malaysia | retrospective study | ACS | NCVD-ACS | 56.72 ± 11.7 | Female 96 (31.7%),  Male 206 (68.2%) | 16 | 211 | yes | 7 | 91 | 30-day mortality |
| Joon-Myoung Kwon 2019 | Korea | retrospective study | AMI | KorMI | T:(64.0 ± 12.8)  V:(63.63 ± 12.6) | Female: T-3521 (29.0%),  V-2992 (27.9%) | 802 | 12152 | yes | 708 | 10723 | 6-month mortality |
| Thomas E Cowling 2021 | UK | retrospective study | AMI | EMR | 70 (58-80) | Male:132,162 (66.0%) | 34520 | 200119 | none | — | — | 1-year mortality |

**Supplementary Table S3.** The key characteristics of the included studies

| **Study ID** | **Country** | **Variable selection method** | **Model type** | **Number of models** | **Number of model variables** | **Model evaluation indicators** | **Outcomes indicators** |
| --- | --- | --- | --- | --- | --- | --- | --- |
| Yudan Wang 2022 | China | univariate+stepwise regression | LR | 1 | 10 | Age,BMI,SBP on admission,HGB,Random blood glucose on admission,EF after PCI,Use aspirin before admission,N/L ratio,Long lesions,TIMI flow grade 0–1 before PCI | C-index  AUC |
| BlancaVázquez 2021 | Mexico | LR/SVM/RF/XGB | LR, SVM, RF, XGB | 8 | Direct inclusion | Demographic(Gender, age, admission type (elective, emergency, urgent), status (divorced, married, single, widow), weight admit)+Vital signs(Heart rate, blood pressure (systolic, diastolic, mean), respiratory rate, oxygen saturation, temperature)+Laboratory results(Troponin T, troponin I, anion gap, albumin, bands, urea, uric acid, creatinine, fibrinogen, sodium, triglycerides, glucose, white blood cells, partial thromboplastin time, neutrophils, lymphocytes, basophils, monocytes, protein creatinine ratio, eosinophils, international normalized ratio, prothrombin time, platelets, potassium, positive end-expiratory pressure, cholesterol (total, hdl, ldl), hemoglobin a1c, hematocrit, hemoglobin, c-reactive, creatine kinase ck, creatine kinase MB)+Hemodynamic(Cardiac out, intracranial pressure, devices beat rate (left, right), pulmonary artery pressure (systolic, diastolic, mean), central venous pressure, ventricular assist device (left, right), pulmonary capillary wedge pressure, mixed venous oxygen saturation, pulmonary artery line, ventricular assist)+Arterial blood gas(Alveolar-arterial gradient, base excess, SO2, PO2, PCO2, Total CO2, chloride, calcium, lactate, FiO2, bicarbonate, PH)+Treatments(Aspirin, clopidogrel bisulfate, enoxaparin, heparin, oral nitrates statins, fibrates, beta-blockers, amiodarone, ace inhibitors, Angiotensin II receptor blockers, insulin, diuretics, calcium antagonist, potassium chloride, oral glucose low drugs, digoxin, dobutamine, dopamine, warfarin, vancomycin)+Procedures(Coronary arteriography using two catheters, injection or infusion of platelet inhibitor, combined right and left heart cardiac catheterization, circulation auxiliary to open-heart surgery, replacement of tracheostomy tube, angiocardiography of left heart structures, insertion of the endotracheal tube, angiocardiography of right heart structures, the extracorporeal implant of pulsation balloon, venous catheterization, coronary arteriography using a single catheter, arterial catheterization, insertion of the temporary transvenous pacemaker system)+Complications(Ventricular fibrillation, ventricular tachycardia, atrial fibrillation, atrioventricular block, angina, left bundle branch block, right bundle branch block, cardiogenic shock, pericarditis, renal failure, hypertension, mitral regurgitation, cardiac arrest, diabetes, congestive heart failure, chronic airway obstruction, aneurysm, cerebrovascular accident, leads (i, ii, iii, v1, v2, v3, v4, v5, v6, avf, avr, avl, f). For STEMI: leads (v1r, v2r), qtc wave. For NSTEMI: leads (lv, l, v), septal rupture, anterolateral, lateral, precordial, inferolateral, anterior, mid-lateral, posterolateral, inferior, hypertrophy, left ventricular, waves (r, qt, inverted t, qrs, rv).) | AUC  Sensitivity  Specificity |
| Roni Shouval 2017 | Israel | the information gain | NB, ADT, LR, PART, RF, AdaBoost | 6 | 54 | creatinine,Killip at admission,DBP,SBP,glucose,age,total cholesterol,hemoglobin,triglycerides,BMI,Number diseased vessels,heart rate,dyspnea at presentation | AUC |
| Syed Waseem Abbas Sherazi 2020 | Korea | None | GBM, DNN, RF, GLM | 4 | 69 | Continuous Variables(Age, BMI, WHR, symptom-to-balloon time (minute), arrival-to-balloon time (minute), SBP, DBP, heart rate, LV ejection fraction in echocardiography, glucose (on admission), creatinine (on admission), maximum CK, maximum CK-MB, maximum troponin I, maximum troponin T, total cholesterol, triglyceride, HDL cholesterol, LDL cholesterol, hsCRP, NT-proBNP, BNP)+Categorical Variables43个(Gender, resuscitation prior to arrival, DOA or not resuscitated state at arrival, symptoms at admission, pain, dyspnea, previous angina before MI symptom, ECG on admission, ischemia location, heart rhythm, history of ischemic heart disease, history of hypertension, history of diabetes mellitus, history of dyslipidemia, history of smoking,  family history of heart disease in the first degree, comorbidities, past regular medication,initial therapeutic strategy, initial therapeutic strategy for STEMI, initial therapeutic strategy for NSTEMI, thrombolysis, why thrombolysis was not performed, PCI, coronary angiographic findings, angiographic findings, target vessel, treated vessel, PCI with stent, if yes target lesion stent type, PCI success, state of revascularization, complications, kind of complications, supportive treatment of complication, echocardiography, stress test, what kind of stress test, result stress test, CABG, electrophysiology study, medical therapy in  hospital, discharge medication, final diagnosis)+Discrete Variables4个(Killip class, lesion type, LV ejection fraction in coronary angiographic findings, mitral regurgitation grade)Note:BMI: body mass index; WHR: waist-to-hip ratio; SBP: systolic blood pressure; DBP: diastolic blood pressure; LV: left ventricular; CK-MB: creatine kinase-muscle brain; DOA: dead on arrival; ECG: electrocardiogram; PCI: percutaneous coronary intervention; CABG: coronary artery bypass grafting; HDL: high-density lipoprotein; LDL: low-density lipopro_x005f_x005f_x005f_x005f_x005f_x005f_x005f_x005f_x005f_x005f_x005f_x005f_x005f_x005f_x005f_x005f_x005f_x005f_x0002_tein; NT-proBNP: N-terminal of the prohormone brain natriuretic peptide; hsCRP: high-sensitivity C-reactive protein; STEMI: ST-segment elevation myocardial infarction; NSTEMI: non-ST-segment elevation myocardial infarction | AUC  Accuracy |
| Konrad Pieszko 2018 | Poland | None | LR, XGBoost, XGBoost+LR | 4 | simplified set of features-23  full set of features-57 | XGBoost simplified set of features:(Neutrophil count,Systolic blood pressure,Creatinine level,Hematocrit level,Age,Troponin elevation ratio,Eosinophil count,BMI(Body mass index),Basophil count,Platelet to lymphocyte ratio,Diastolic blood pressure,monocyte count,Platelet count, Fibrinogen level,LDL level,Weight,Height,HDL level,Mean platelet volume,Neutrophil to lymphocyte ratio,Heart rate,Red cell distribution width,Lymphocyte count);DRSA-BRE full set of features: (Impaired fasting glycaemia,LDL level,Mean cell volume,Fibrinogen level,Impaired glucose tolerance,Type 1 diabetes,Platelet to lymphocyte ratio,Potassium level,Triglycerides level,HDL level,Platelet count,BMI(Body mass index),TSH level(Thyroid Stimulating Hormone level),Total cholesterol level,Aspartate aminotransferase level,Height,Creatinine level,Lymphocyte count,Eosinophil count,Neutrophil to lymphocyte ratio,Monocyte count,Weight,Glomerular filtration rate,Red cell distribution width,Thrombin time,Hypertension,Hematocrit level,History of pulmonary disease,Hemoglobin level,Basophile count,History of renal failure,Urea level,Mean platelet volume,History of stroke,Sodium level,Alanine aminotransferase level,Activated partial thromboplastin time,History of heart failure,Heart rate,Sex,History of CABG,History of myocardial infarction,Family history of coronary heart disease,active Smoker,Former smoker,history of PTCA, No history of smoking,Troponin elevation ratio,Neutrophil count, History of coronary artery disease,History of peripheral artery disease,Age,Systolic blood pressure,No Diabetes,Diastolic blood pressure,prothrombin time, Type 2 diabetes);DRSA-BRE simplified set of features: (LDL level,Monocyte count,Platelet to lymphocyte ratio,Lymphocyte count,Fibrinogen level,HDL level,Platelet count,BMI(Body mass index), Hematocrit level,Height,Red cell distribution width,Basophil count,Eosinophil count,Creatinine level,Weight,Neutrophil to lymphocyte ratio,Mean platelet volume,Heart rate,Age,Systolic blood pressure,Neutrophil count,Troponin elevation ratio,Diastolic blood pressure) | AUC  Sensitivity  Specificity  Accuracy |
| Konrad Pieszko 2019 | Poland | None | gradient-boosted trees | 3 | 19 | troponin elevation ratio,Neutrophil to lymphocyte ratio(NLR),Platelet to lymphocyte ratio(PLR),Red cell distribution width(RDW),CRP,platelet count,creatinine level,hemoglobin level,mean cell volume,sodium level,prothrombin time,fibrinogen level,age,neutrophil count,body mass index(BMI),systolic blood pressure,diastolic blood pressure,heart rate,sex | AUC |
| Jacek T Niedziela 2021 | Poland | univariate | ANN, LR | 2 | 42 | age, sex, body mass index, systolic blood pressure on admission, time from the hospital admission to PCI, heart rhythm on admission, bundle branch block, the principal symptom of ACS, the Killip class on admission, a history of diabetes, coronary artery disease, heart failure, stroke, chronic kidney disease, peripheral artery disease, chronic obstructive pulmonary disease or smoking, in-hospital treatment (β-blockers, angiotensin-converting enzyme inhibitors [ACEI], diuretics, insulin, inotropes, glycoprotein (GP) IIb/IIIa, intra-aortic balloon pump use, type of stent — metal, drug-eluting stent, or none), thrombolysis in  myocardial infarction (TIMI) score after the PCI, in-hospital complications (pulmonary edema, cardiogenic shock, bleeding requiring blood transfusion, stroke, cardiac arrest), the length of hospital stay, the latest available New York Heart Association class, left ventricular ejection fraction, and treatment recommended at discharge (β-blockers, ACEI, statins, diuretics, insulin, acetylsalicylic acid, second antiplatelet drug, low-molecular-weight heparin). | AUC |
| Paul D Myers 2017 | USA | None | ANN, LRHx, LRST, LRHx+ST, LRHx+MV, LRHx+HRV, LRHx+DC, RNN | 8 | LRHx-7, LRST-4, LRHx+ST-11, LRHx+MV-8, LRHx+HRV-8, LRHx+DC-8, RNN-1, ANN-8 | LRHx:age, gender, whether the patient was a smoker at the time of enrollment, history of hypertension, history of diabetes, previous myocardial infarction (MI), and previous angiography;  LRST:corresponding to the mean and standard deviation of the first two coefficients representing the level and slope of the ST segments of the first 50 beats from the Holter; LRHx+ST:age, gender, whether the patient was a smoker at the time of enrollment, history of hypertension, history of diabetes, previous myocardial infarction (MI), and previous angiography,corresponding to the mean and standard deviation of the first two coefficients representing the level and slope of the ST segments of the first 50 beats from the Holter; LRHx+MV:age, gender, whether the patient was a smoker at the time of enrollment, history of hypertension, history of diabetes, previous myocardial infarction (MI), and previous angiography,Morphologic Variability (MV); LRHx+HRV:age, gender, whether the patient was a smoker at the time of enrollment, history of hypertension, history of diabetes, previous myocardial infarction (MI), and previous angiography,Heart Rate Variability (HRV); LRHx+DC:age, gender, whether the patient was a smoker at the time of enrollment, history of hypertension, history of diabetes, previous myocardial infarction (MI), and previous angiography,Deceleration Capacity (DC) ; RNN:the first two Legendre polynomial coefficients; ANN:age, gender, whether the patient was a smoker at the time of enrollment, history of hypertension, history of diabetes, previous myocardial infarction (MI), and previous angiography,the first two Legendre polynomial coefficients | AUC |
| Woojoo Lee 2021 | Korea | Lasso/Ridge/Elastic net/RF/SVM/XGBoost | LR+Lasso, LR+Ridge, LR+Elastic net, RF, SVM, XGBoost | 36 | STEMI:in-hospital mortality-XGBoost-20  3-month mortality-RF-44  1-year mortality-Lasso-11  NSTEMI: in-hospital mortality-Lasso-20  3-month mortality-Elastic net-19  1-year mortality-Elastic net-27 | STEMI:in-hospital mortality-XGBoost:Cardiogenic shock,Creatinine,Hemoglobin,Systolic BP,Heart rate,Heart failure,Cardiac arrest before ED arrival,Chest pain,Diastolic BP,Height,Loss of awareness,Weight,Dyspnea,Troponin,Smoking,Atrial fibrillation at arrival,Sex,Vertigo and systemic weakness,Epigastric pain,Hypertension;3-month mortality-RF:LV ejection fraction,Heart rate,Bottom hemoglobin,Peak creatinine,Hemoglobin,Creatinine,Weight,Systolic BP,Diastolic BP,Door-to-Balloon time,Height,Smoking,Culprit lesion,Stenosis of pLAD,Hypertension,Dyspnea,Sweat,Sex,Diabetes mellitus,Cardiac arrest before ED arrival,Heart failure during admission,Atrial fibrillation during admission,Heart failure,Chest pain,Complete revascularization,Troponin,Vertigo and systemic weakness,Radiating pain,Cardiogenic shock during admission,Previous stroke,Loss of awareness,Stent insertion,Atrial fibrillation at discharge,Cardiogenicshock,Dyslipidemia,Epigastric pain,Previous PCI,Previous MI,Three vessel disease,Atrial fibrillation at arrival,Stenosis of the left main artery,Cardiac arrest during admission,Peak troponin; 1-year mortality-Lasso:Atrial fibrillation during admission,Smoking,Peak creatinine,Stenosis of pLAD,Age,Heart rate,LV ejection fraction,Sweat,Bottom hemoglobin,Hemoglobin,Chest pain; NSTEMI:in-hospital mortality-Lasso:Cardiogenic shock,Cardiac arrest before ED arrival,Epigastric pain,Heart failure,Dyspnea,Previous stroke,Diabetes mellitus,Hypertension,Creatinine,Age,ECG,Heart rate,Diastolic BP,Systolic BP,Hemoglobin,Smoking,Atrial fibrillation at arrival,Sweat,Chest pain,Radiating pain;3-month mortality-Elastic net:Atrial fibrillation during admission,Vertigo and systemic weakness,Diabetes mellitus,Heart failure during admission,Peak creatinine,Weight,Dyspnea,Cardiogenic shock during admission,Three vessel disease,Age,Heart failure,Heart rate,Diastolic BP,LV ejection fraction,ECG,Hemoglobin,Cardiogenic shock,Bottom hemoglobin,Dyslipidemia;1-year mortality-Elastic net:Previous MI,Three vessel disease,Previous stroke,Dyspnea,Atrial fibrillation during admission,Heart failure during admission,Diabetes mellitus,Cardiogenic shock during admission,Troponin,Peak creatinine,Atrial fibrillation at discharge,Heart failure,Age,Height,Atrial fibrillation at arrival,Heart rate,Systolic BP,Diastolic BP,LV ejection fraction,Weight,Hemoglobin,Dyslipidemia,Bottom hemoglobin,Chest pain,ECG,Cardiogenic shock,Cardiac arrest before ED arrival. | AUC  Specificity  Sensitivity  Accuracy |
| D J Kurz 2009 | Switzerland | C4.5 | AODE algorithm | 3 | 7 | age,Killip class,systolic blood pressure,heart rate,pre-hospital cardiopulmonary resuscitation,history of heart failure,history of cerebrovascular disease. | C-index |
| Jun Ke 2022 | China | univariate+stepwise regression | LR, GBDT, RF, SVM | 4 | LR-10, GBDT-10, RF-10, SVM-Not mentioned | LR: age,NSTEMI,Killip III,Killip IV,D-Dimer,cTnI,CK,NT-proBNP,HDL-C,Statins; GBDT:NT-proBNP,D-dimer,Killip,cTnI,LDH,LVEF,diagnosis,age,HDL-C,CK; RF:Killip,D-dimer,NT-proBNP,LVEF,LDH, cTnI,diagnosis,age,LDL-C, HDL-C. SVM:Not mentioned | AUC  Specificity  Sensitivity |
| Jaroslav Hubacek 2006 | Canada | univariate+stepwise regression | LR | 1 | 11 | Extended MM:AMI to PCI within 24h,Indication for PCI (MI),Cardiogenic shock,Increased creatinine levels,Cardiac arrest,number of diseased vessels,Age(70-79､≥80),EF(<50%､Missing､Unable to do),Thrombus,Peripheral vascular disease,Female sex MM:age(70-79､≥80), female sex, cardiogenic shock, MI within 24 h, number of diseased vessels, visible thrombus, creatinine>1.5 mg/dL, history of cardiac arrest, peripheral vascular disease, EF<50%(decreased ejection fraction) | C-index |
| Amir Hadanny 2021 | Israel | a feature selection algorithm | RF | 2 | RF-32, RF-Simple-10 | RF:Age,Male,Heart Rate,MAP,BMI,Total Cholesterol,Glucose,Hemoglobin,Creatinine,Time from Onset to PCI,Past MI,Chronic heart failure,Past Revascularization,Past Stroke,Family history of MI,Chronic renal failure,PVD,Diabetes Mellitus,Hypertension,Killip class at admission,Chronic beta blockers,Chronic antiplatelet drugs,Any smoking,Chronic ACE-I/ARBB,Typical angina presenting symptom,Normal Sinus Rhythm,CPR/DCS,Aspirin before admission,Clopidogrel before admission,Chronic Hyperlipidemia drugs,Anterior MI. RF-Simple:Creatinine,MAP,Killip class at admission,Glucose,Age,Total Cholesterol,Hemoglobin,Heart Rate,BMI,Time from Onset to PCI | AUC |
| Vasim Farooq 2013 | Netherlands | LR | LR | 1 | 10 | "Core" Model:anatomical SYNTAX score,age,creatinine clearance,left ventricular ejection fraction; Extended model:anatomical SYNTAX score,age,creatinine clearance,left ventricular ejection fraction,presentation(Stable､UA､NSTEMI ､STEMI), body mass index (BMI),peripheral vascular disease,diabetes,previous MI,smoking | AUC |
| Irene R Dégano 2015 | Spain | stepwise LR | LR | 1 | 8 | Clinical AMI modle:sex,age, hypertension,diabetes,ACS type,CCU/ICU,cardiogenic shock/acute pulmonary oedema,renal failure on admission; Administrative AMI modle:sex,age,hypertension,diabetes,ACS type,CCU/ICU,past history of CV disease; Clinical PCI modle:diabetes,past history of CV disease,ACS type,catheterization laboratory,university hospital,cardiogenic shock/acute pulmonary oedema,renal failure on admission; Administrative PCI modle:diabetes,past history of CV disease,ACS type,catheterization laboratory,university hospital,age,hypertension; | C-index |
| Fabrizio D'Ascenzo 2021 | Italy | None | Adaboosting | 1 | 25 | age,sex,diabetes,hypertension,hyperlipid­aemia,peripheral artery disease,estimated glomerular filtration rate,previous myocardial infarction,previous percutaneous coronary intervention,previous coronary artery bypass graft,previous stroke,previous bleeding,malignancy,ST­segment elevation myocardial infarction [STEMI] presentation,haemoglobin,and left ventricular ejection fraction [LVEF]),five thera­peutic variables (treatment with β blockers,angiotensin­  converting enzyme inhibitors or angiotensin­receptor blockers,statins,oral anticoagulation,and proton­pump inhibitors,multivessel disease and complete revascularisation,vascular access and percutaneous coronary inter vention with drug­eluting stent | AUC |
| Shao-di Yan 2016 | China | univariate+stepwise regression | LR | 1 | 9 | serum calcium at admission,age,heart rate,systolic blood pressure,serum creatinine (SCr) concentration,Killip class,ST‐segment deviation,elevated cardiac enzymes, and cardiac arrest | AUC |
| Francisco Valente 2021 | Portugal | univariate+stepwise regression | LR, ANN | 2 | 9 | diagnosis,age,antecedent of stroke/transient ischemic attack,systolic blood pressures,heart rate,Killip class,left ventricular ejection fraction,glucose,hemoglobin. | AUC |
| Ana T Timóteo 2014 | Portugal | univariate+stepwise regression | LR | 1 | 9 | admission blood glucose,age,heart rate,systolic blood pressure,serum creatinine (SCr) concentration,Killip class,ST‐segment deviation,elevated cardiac enzymes, and cardiac arrest | AUC |
| Ana T Timóteo 2015 | Portugal | univariate+stepwise regression | LR | 1 | 9 | RDW/PDW,age,heart rate,systolic blood pressure,serum creatinine (SCr) concentration,Killip class,ST‐segment deviation,elevated cardiac enzymes,and cardiac arrest | AUC |
| Liao Tan 2021 | China | Lasso | LR | 1 | 14 | age,history of peripheral vascular disease,atrial fibrillation,cardiogenic shock and cardiac arrest,the use of norepinephrine,urine output,white blood cell (WBC), hemoglobin(Hb),red blood cell(RBC),red cell distribution width(RDW),glucose,bicarbonate and magnesium. | C-index  AUC |
| Paul D Myers 2019 | USA | Lasso | RLRVI | 1 | 19 | Age,Admission weight,Congestive heart failure,Peripheral artery disease,Renal insufciency,Systolic blood pressure,Pulse,Killip class,Cardiac arrest,ST segment deviation,Warfarin,Medications,pre-hospital or within 1st 24hours, Statin,Diuretic, Insulin, IV inotropic agent , Oral beta blocker, IV beta blocker, Initial creatinine, Initial positive enzymes | C-index  AUC |
| Rohan Khera 2021 | USA | None | LR, XGBoost, NN, meta-classifier model | 4 | — | Demographics(Age,Weight,BMI,Sex,Race,Hispanic origin)+Medical History(History of diabetes mellitus,Diabetes control,History of hypertension,History of dyslipidemia,Current/recent smoker,Current dialysis,Chronic lung disease,History of MI,History of heart failure,Prior PCI,Prior CABG,History of atrial fibrillation,Prior cerebrovascular disease,Prior peripheral artery disease,Prior stroke,Prior transient ischemic attack)+Presentation(After Cardiac Arrest,In Cardiogenic shock,In heart failure,Heart rate,SBP)+Presentation ECG(ST-elevation myocardial infarction,New or presumed new ST-segment depression,New or presumed new T-wave inversion,Transient ST-segment elevation < 20 minutes,ST elevation,Left bundle branch block,Isolated posterior MI)+Home Medications(Aspirin,Clopidogrel,ACE inhibitor,Angiotensin receptor blocker,Beta blocker,Statin,Non-statin lipid-lowering agent,Prasugrel,Warfarin,Aldosterone blocking agent)+Initial Laboratory Tests(Initial CKMB collected,Initial Troponin collected,Initial Creatinine collected,Initial Hemoglobin collected,Lipid panel collected,Initial BNP collected,Initial pro-BNP collected,Troponin Ratio,Creatinine,Creatinine Clearance,Hemoglobin) | C-index  AUC |
| Zhengxing Huang 2018 | China | None | ANN | 1 | — | Age,Creatinine et al | AUC |
| Dongze Li 2021 | China | univariate+stepwise regression | LR | 1 | 4 | age,heart rate,BMI and Killip class | AUC |
| Truong H Hoang 2021 | Russia | univariate+stepwise regression | LR | 1 | 4 | Hemoglobin at admission,Presence of ST elevation,**Prior myocardial infarction,Charlson comorbidity index** | C-index  AUC  Specificity  Sensitivity |
| Jia Zhao 2021 | China | None | LR, SVM, DT, RF | 4 | 16 | simple:Sex,Age,Consciousness,Respiration,Pulse,Heart rate,Systolic blood pressure,Diastolic blood pressure,Killip class,Cardiac troponin I,Time from symptom to first medical contact,Prehospital mode of transport,Heart failure; full:Sex,Age,Consciousness,Respiration,Pulse,Heart rate,Systolic blood pressure,Diastolic blood pressure,Killip class,Cardiac troponin I,Time from symptom to first medical contact,Prehospital mode of transport,Heart failure,Thrombolysis,Reperfusion treatment,TIMI flow after intervention; | AUC  Sensitivity  Specificity  Accuracy |
| Iman Yosefian 2015 | Iran | None | Saturated tree, Pruned tree, RSF | 3 | 11 | age,sex,hypertension disease,hyperlipidemia,history of ischemic heart disease at admission,diabetes,smoking status,family history of AMI disease,Q wave status, streptokinase treatment,and intervention(angioplasty,pacemaker surgery,bypass surgery,and drug therapy). | C-index |
| Ni Wang 2022 | China | None | KNN, LR, RF, LSTM, RNN, KNND, KNNH, KNNE, KNNEH, KNNED | 10 | — | — | AUC |
| John Wallert 2017 | Sweden | LR, C5.0, RF, SVM | LR, C5.0, RF, SVM | 4 | 39 | LR:Age,diacharge Statins,heart rate,Diabetes,Smoking,systolic blood pressure,diacharge ECG rhythm,history of taking Beta blockers,weight,diacharge ACE inhibitors,diacharge other antiplatelet,previous stroke,CCU No reperfusion,CCU QRS Normal,history of taking ACE inhibitors C5.0:systolic blood pressure,Age,CCU No Pulmonary rales,weight,diacharge other antiplatelet,diacharge Statins,diacharge ECG rhythm,previous stroke,heart rate,Diabetes,history of taking Beta blockers,intake Dyspnea,history of taking ACE inhibitors,Smoking,CCU QRS Left bundle branch block RF:Age,heart rate,weight,CCU Troponin,systolic blood pressure,diacharge Statins,diacharge other antiplatelet,intake Chest pain,Smoking,Diabetes,history of taking Beta blockers,Ambulance to CCU,diacharge ACE inhibitors,diacharge ECG rhythm SVM:Age,diacharge Statins,weight,heart rate,diacharge other antiplatelet,intake Chest pain,CCU No Pulmonary rales,CCU Primary PCI,CCU sinus,CCU QRS Normal,systolic blood pressure,sex,diacharge ACE inhibitors,CCU ST-elevation,Smoking | AUC  Sensitivity  Specificity  Accuracy |
| Alessia Vignoli 2019 | Italy | univariate+stepwise regression | RF | 1 | — | — | AUC  Sensitivity  Specificity  Accuracy |
| Yu Tan 2021 | China | univariate+stepwise regression | LR | 1 | 10 | TMAO,MPO,age,heart rate,systolic blood pressure,initial serum creatinine,Killip class,cardiac arrest on admission,elevated troponin I level,and ST-segment deviation | AUC |
| Ewout W Steyerberg 2014 | Netherlands | — | LR | 1 | — | age,Killip class,systolic blood pressure,and heart rate et al. | C-index |
| Salman I 2019 | Czech Republic | Lasso | NB, C4.5, LOG.REG, NB-tree, BN.K2, BN, TAN | 6 | — | — | AUC  Accuracy |
| Piros, P 2019 | Hungary | Direct inclusion | LR, DT, ANN | 3 | 15 | Gender,Age,Prior Myocardial Infarction,Previous Heart failure,Hypertension,Prior Stroke,Diabetes mellitus,Peripheral artery disease,Hyperlipidaemia,Smoking (current + former),Prehospital reanimation,Cardiogenic shock,Percutaneous Coronary Intervention (PCI) during hospital stay,Creatinine,ACS type | AUC |
| D. Mendes 2018 | Portugal | Direct inclusion | DT | 1 | 8 | Age,Creatinine,Systolic Blood Pressure,Heart Rate,Killip class,ST-segment deviation,Elevated cardiac markers,Cardiac arrest at admission | Sensitivity  Specificity  Accuracy |
| Hend Mansoor 2017 | USA | univariate + stepwise regression | LR, RF, RF | 3 | LR-11, Full model for RF-32, Reduced model for RF-17 | LR:age >70 years,number of chronic conditions ≥7,smoking,absence of HTN as well as dyslipidemia,family history of coronary artery disease (CAD),previous history of CAD,lack of angiography during the hospitalization,lack of PCI during the hospitalization,renal failure, and cardiogenic shock.  Full model for RF:Age,Race,Expected primary payer,Number of days from admission to first procedure,Weekend admissions,Median household income,Angiography during hospitalization,PCI during hospitalization,Division of hospital,Alcohol abuse,Deficiency anemia,Rheumatoid arthritis/collagen vascular diseases,Congestive heart failure,Chronic pulmonary disease,Coagulopathy,Depression,Diabetes,Diabetes with chronic complications,Drug abuse,Hypertension,Liver disease,Hypothyroidism,Obesity,Peripheral vascular disorders,Pulmonary circulation disorders,Renal failure,Valvular heart disease,CAD,Family History of CAD,History of PCI,Dyslipidemia,Smoking,Cardiogenic shock; Reduced model for RF:Hospital division,Cardiogenic shock,Patient’s zip code,Race,Number of days from admission to first procedure,Expected primary payer,Weekend admissions,Angiography during hospitalization,Diabetes Mellitus,PCI during hospitalization,CAD,Hypertension,Chronic lung disease,Dyslipidemia,Deficiency anemia,Number of chronic conditions,Age>70 years; | C-index  AUC  Accuracy |
| Yi-Ming Li 2020 | China | RF+XGBoost | LR, NB, KNN, DT, RF, XGBoost | 6 | 15 | New York Heart Association (NYHA) Classification at discharge,heart failure at admission,heart rate,age,left ventricular ejection fraction,serum cystatin,initial BNP, Platelet,Fibrinogen,Blood creatinine,blood glucose,systolic blood pressure,diastolic blood pressure,total bilirubin,blood urea nitrogen,and revascularization type. | AUC  Sensitivity  Specificity  Accuracy |
| Christien Kh Li 2020 | China | stepwise LR | ANN | 1 | 4 | neutrophil-to-lymphocyte ratio,peripheral vascular disease,age and creatinine | C-index |
| Han Cheol Lee 2020 | Korea | PCA+RFE | DT+LR+DN | 1 | 19 | age,left ventricular ejection fraction during hospitalization,initial Killip class,body mass index(BMI),initial serum creatinine,high-density lipoprotein-C,PCI,SBP,C-reactive protein,peak CK-MB,heart rate,peak Troponin I,cardiogenic shock during hospitalization,initial serum glucose,initial serum total cholesterol,low-density lipoprotein-C,triglyceride | AUC |
| Meng-Hsuen Hsieh 2019 | China | None | ANN, DT, LDA, LR, NB, SVM | 6 | 24 | Culprit lesion,Multi-vessel PCI,Age,Gender,Comorbidity(Hyperlipidemia､Hypertension､Diabetes mellitus､Chronic obstructive pulmonary disease､Heart failure､Stroke､Atrial fibrillation､Obesity､Chronic kidney disease/ESRD､PAOD､History of PCI､ History of CABG､ History of IABP), Medications(Aspirin､Warfarin､Clopidogrel),30-day postoperative complication(Upper gastrointestinal bleeding､Acute kidney injury､Arrhythmia､Admitted to ICU) | AUC |
| Yuhei Goriki 2020 | Japan | univariate+stepwise regression | LR | 1 | 5 | Platelet <15 × 104/μL,BS ≥200 mg/dL,eGFR <45 mL/min/1.73m2,Albumin ≤3.5mg/dL,High-sensitivity troponin I >1.6 ng/dL (normal upper limit × 50) | AUC |
| Nan Gao 2020 | China | Lasso | LR | 1 | 14 | sex,Killip classification,left main coronary artery disease (LMCAD),grading of thrombus,TIMI classification,slow flow,application of IABP,administration of β-blocker, ACEI/ARB,symptom-to-door time (SDT),symptom-to-balloon time (SBT),syntax score,left ventricular ejection fraction (LVEF),and CK-MB peak. | AUC  Sensitivity  Specificity |
| Rui Fu 2018 | China | univariate+stepwise regression | LR | 1 | 11 | age,body mass index,systolic blood pressure,Killip classification,cardiac arrest,electrocardiogram ST-segment depression,serum creatinine,white blood cells, smoking status,previous angina, and previous percutaneous coronary intervention. | C-index |
| Victoria A Brazhnik 2021 | Russia | CART | DT | 1 | 39 | Age,uric acid level,an option of antithrombotic therapy,a history of chronic heart failure,SBP,Hb,DBP,alcohol abuse,glucose,history of CAD,smoking,creatinine,WBC,acute HF,HR at the discharge,Max SBP,Hyperlipidemia,aortic stenosis | AUC  Sensitivity |
| Theodor Baars 2018 | Germany | ensemble feature selection approach | LR | 1 | 5 | age,CRP,eGFR,myoglobin,and urea | AUC |
| Firdaus Aziz 2021 | Malaysia | RF, SVM, LR | RF, SVM, LR, SVMvarImp-SBE-SVM | 4 | in-hospital mortality-15  30-day mortality-13  1-year mortality-12 | in-hospital mortality:Age,Family history of premature CVD,Chronic renal disease,Heart rate,Systolic blood pressure,Diastolic blood pressure,Killip class,Fasting blood glucose,ECG-type bundle branch block,ECG- location lateral lead,Cardiac catheterization,Beta blockers,Statin,Diuretics,Oral hypoglycaemic agent 30-day mortality:Age,Race,Family history of premature CVD,Heart rate,Killip class,HDL,Fasting blood glucose,Cardiac catheterization,PCI,ASA,Beta blockers,Diuretics,Oral hypoglycaemic agent 1- year mortality:Age､Smoking status､Hypertension､Diabetes､Heart rate､Systolic blood pressure､Killip class､Fasting blood glucose､PCI､ACE inhibitor､Diuretics､Insulin | AUC |
| Aziida, N 2021 | Malaysia | RF, Boruta | Rfimp, BorutaLR | 2 | RFimp-SBS-EN-8  Boruta-SBS-LR-3 | RFimp-SBS-EN:Age,TC,Tg,Troponin,creatinine,HDL,eGFR,CK Boruta-SBS-LR:Age,TC,Tg | AUC |
| Joon-Myoung Kwon 2019 | Korea | None | RF, LR, ANN | 3 | 13 | age,sex,body mass index,cardiac arrest before visit,systolic blood pressure,heart rate,Killip class,creatinine kinase-muscle/brain (CK-MB),glucose,C-reactive protein (CRP),creatinine,low-density lipoprotein,and elevation of the ST segment | AUC |
| Thomas E Cowling 2021 | UK | None | LR, Boosted trees | 2 | 3 | age,sex,and socioeconomic status | C-index |

**
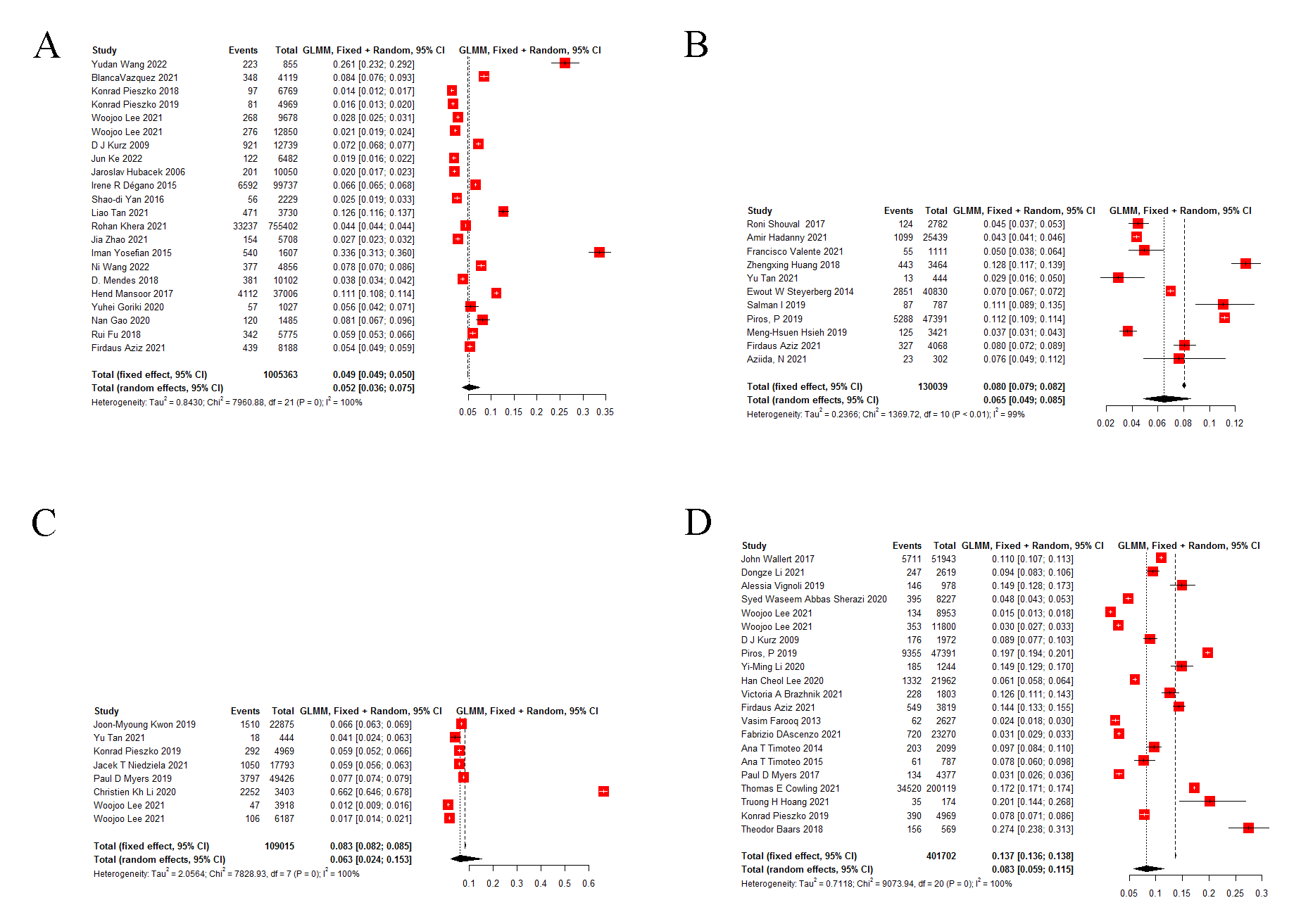
**

**Supplementary Figure S1.** A. in-hospital mortality; B. 30-day mortality; C. 3- or 6-month mortality; D. 1-year or more mortality.


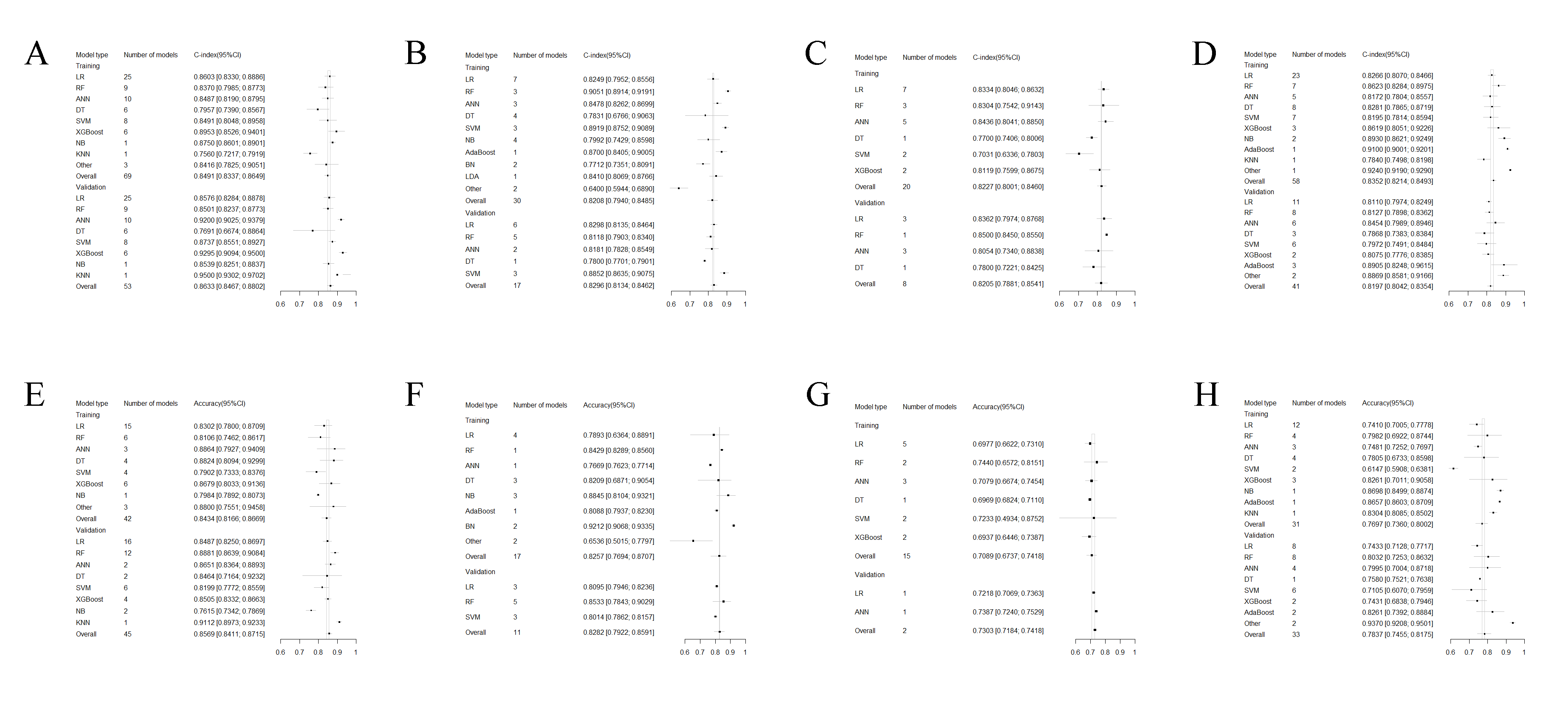


**Supplementary Figure S2**. A. C-index for in-hospital mortality; B. C-index for 30-day mortality; C. C-index for 3- or 6-month mortality; D. C-index for 1-year or more mortality; E. Accuracy for in-hospital mortality; F. Accuracy for 30-day mortality; G. Accuracy for 3- or 6-month mortality. H. Accuracy for 1-year or more mortality.

**Biostatistics Review Certificate**

**Name of Journal:** European Journal of Medical Research

**Title:** The predictive value of machine learning for mortality risk in patients with acute coronary syndromes: a systematic review and meta-analysis

**Authors List:** Xiaoxiao Zhang (MD), Xi Wang (MM), Luxin Xu (MM), Jia Liu (MD), Peng Ren (MD), Huanlin Wu (MD)

**Correspondence to:** Huanlin Wu; E-mail: wuhuanlinboshi@aliyun.com

**The statistical methods of this study were reviewed by a member of the STATRAY from Yanchang Science and Technology Co., Ltd of China.**

Name: Banghong Chen

Signature:
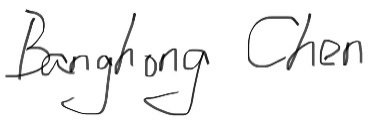


Contact information: Email: [cbh321628@126.com](mailto:cbh321628@126.com)
